# Magneto-Structural Correlations in Coordination Polymers Based on Formate Ligand and Transition Metal Cations

**DOI:** 10.1021/acs.inorgchem.4c04737

**Published:** 2025-04-10

**Authors:** Francisco Rubio-Sepúlveda, Alicia Manjón-Sanz, Laura Cañadillas-Delgado, José Alberto Rodríguez-Velamazán, Lukas Keller, Denis Sheptyakov, Diego Venegas-Yazigi, Verónica Paredes-García, Javier Campo

**Affiliations:** a Facultad de Química y Biología, Departamento de Química de los Materiales, Universidad de Santiago de Chile, Santiago 9170022, Chile; b Instituto de Nanociencia y Materiales de Aragón, CSIC - Universidad de Zaragoza, Zaragoza 50009, Spain; c Centro para el Desarrollo de la Nanociencia y Nanotecnología CEDENNA, Santiago 9170022, Chile; d Neutrons Scattering Div, Oak Ridge Natl Lab, Oak Ridge, Tennessee 37831, United States; e Institut Laue Langevin (ILL), CS 20156, Cedex 9, Grenoble 38042, France; f Laboratory for Neutron Scattering and Imaging, Paul Scherrer Institut, Villigen 5232, Switzerland; g Facultad de Ciencias Exactas, Departamento de Ciencias Químicas, Universidad Andres Bello, Santiago 8370146, Chile

## Abstract

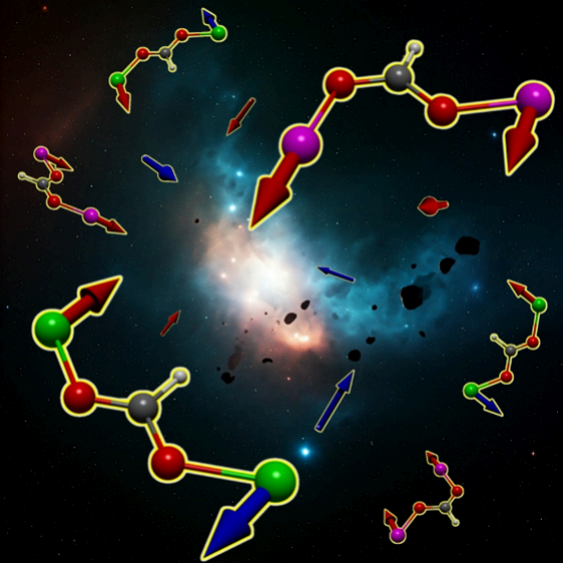

We present five three-dimensional (3D) coordination polymers (CPs) based on the formate ligand, [NaM(HCOO)_3_(H_2_O)]_*n*_ with M = Co^2+^ and Ni^2+^ and [KM(HCOO)_3_]_*n*_ with M = Mn^2+^, Co^2+^, and Ni^2+^, introducing three new nuclear structures with the *P*2_1_ space group for [NaCo(HCOO)_3_(H_2_O)]_*n*_ and [NaNi(HCOO)_3_(H_2_O)]_*n*_, and *P*6_3_22 Sohncke SG with chiral nuclear structure for [KNi(HCOO)_3_]_*n*_, along two centric *C*2/*c* isomorphs [KMn(HCOO)_3_]_*n*_ and [KCo(HCOO)_3_]_*n*_. Magnetic measurements indicate that antiferromagnetic interactions predominate in the five CPs, with averaged antiferromagnetic *zJ*/*k*_B_ mean values from −1.18 to −94.9 K. Moreover, magnetic long-range order (LRO) at low temperatures is evidenced by the magnetic susceptibility and heat capacity measurements. Furthermore, single-crystal and powder neutron diffraction experiments were performed to elucidate the magnetic structure, confirming the antiferromagnetic ordering with possible spin canting, thus understanding these systems’ magnetic exchange pathway topology.

## Abstract 2

We report five three-dimensional formate-based coordination polymers (CPs) with long-range magnetic ordering. A magneto-thermal study reveals general antiferromagnetic behaviors corroborated by neutron diffraction experiments, which evidenced global AF magnetic structures for the five CPs.

## Introduction

Materials based on covalently extended coordination entities, or CPs, have been widely studied for more than half a century because they can present attractive properties in catalysis, magnetism, and nonlinear optical properties (NLO), among others.^1−6^ Furthermore, the rational design of CPs allows us to correlate the structural features with the target properties, making this an active and attractive field of research.^7−10^ In this sense, nuclear chirality (absence of any mirror symmetry) is a structural characteristic that can raise interesting material properties and enhance other ones already present in the material, such as catalysts in enantioselective synthesis^11−13^ and NLO activity used as sensors.^14−17^

On the other hand, magnetic chirality refers to the chiral symmetry of magnetic structures, inducing a preferred rotation of the magnetization.^18^ Although it could be present in centrosymmetric nuclear structures,^19−22^ magnetic chirality can also be induced from chiral or noncentrosymmetric nuclear structures as this symmetry feature promotes antisymmetric magnetic interactions (Dzyaloshinskii-Moriya interactions (DMIs)), which play a crucial role in noncollinear magnetic orderings.^18,23−25^ The interplay between nuclear and magnetic chirality has been previously studied, with the aim of developing new materials for data storage, spintronic, and quantum computing.^26−28^

**Figure 1:**
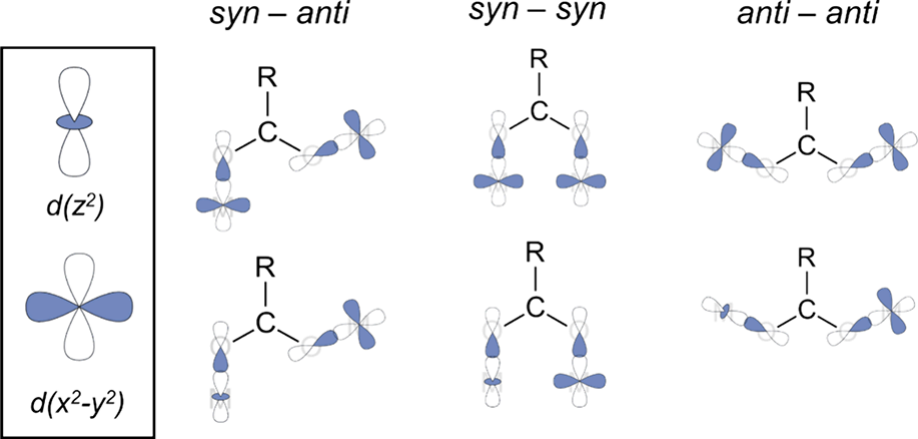
d_*x*_^2^_–*y*_^2^ and d_*z*_^2^ orbital disposition in some carboxylate bridge modes.

The choice of the ligand is a crucial step in the rational design to achieve the desired symmetry features and properties. Regarding this point, carboxylate ligands are attractive candidates in the design of CPs as they can act as both chelating and bridging ligands, affording one-, two-, or 3D networks. Additionally, they can be present simultaneously, conferring a nonlinear linking character to the ligand, therefore adding more complexity to the nuclear structure. More about the bridging character, this group can link two or more cations in different ways, and it has been reported that some of these bridging configurations can be correlated to the nature of magnetic interaction between cations in octahedral symmetry where the magnetic orbitals are the *e*_g_ level, as has been reported for Cu^2+^ compounds.^29−31^ Herein, the *syn–syn* and *anti–anti* modes are related to antiferromagnetic interactions as they promote direct overlapping, while the *syn–anti* is associated with ferromagnetic coupling, as in this case, the direct overlapping is inhibited by the geometry of the bridge (Figure 1).

This magneto-structural correlation can also be evidenced in the work of Jo̷rgensen et al.,^32^ which reports the magnetic structure of the [Ni(HCOO)_2_(H_2_O)_2_]_*n*_ 3D CPs elucidated by neutron diffraction.

The formate anion is an interesting building block when it comes to designing magnetic CPs because it is the smallest of its kind, with R = H. This structural character results in compressed frameworks and, thus, shorter distances between spin centers, which can enhance the magnetic interactions, achieving long-range magnetic ordering (LRO).

Moreover, the literature indicates that the arrangement of this organic ligand around the metal cations can lead to acentric or even polar nuclear structures.^33−38^ An interesting example of this ligand’s structural plasticity is Duan et al.’s work^39^ about the structural transition of the polymorph [KCo(HCOO)_3_]_*n*_ from the Sohncke *P*6_3_22 space group to the *C*2/c centric system.

Also, the effect of cation size on the crystallographic phase evolution in [*AB*(HCOO_3_)]_*n*_ (*A* = Rb, Cs; *B* = Mn, Co, Ni) and its magnetic properties have been reported by Bovill and Saines.^40^ Wang et al. studied the derivative with *A* = NH_4_.^41^

On the other hand, the absence of an inversion center in the structure opens the possibility of presenting DMIs between the magnetic cations. In this regard, Ichiraku et al.^42^ anticipated, by using magnetometry characterization, a chiral soliton lattice originated by a DMI in the {[NH_4_][Mn(HCOO)_3_]}_*n*_ compound. To contribute to the characterization of these magnetic materials, neutron diffraction is a powerful tool for elucidating their magnetic structures. To this date, several examples of successful magnetic structure determinations on formate-based CPs can be found, such as {[CH_3_NH_3_][Co(HCOO)_3_]}_*n*_ in the work of Mazzuca et al.^43^ or the incommensurate magnetic structure of {[CH_3_NH_3_][Ni(HCOO)_3_]}_*n*_ reported by Cañadillas-Delgado et al.^44^ Greenfield et al.^45^ reported the magnetic study of the formate-based CP [Co_3_(HCOO)_5_Cl(H_2_O)_2_]_*n*_, where powder neutron diffraction determined a complex arrangement of three helical chains with a triangular arrangement, which led to a homospin topological ferrimagnet and, therefore, to understanding the isothermal magnetization data.

Considering the possibility of synthesizing CPs with acentric or chiral nuclear structures, which could lead to antisymmetric interactions in formate-based frameworks with magnetic LRO, herein, we report the characterization of five 3D CPs based on the formate ligand and Mn^2+^, Co^2+^, and Ni^2+^ cations. Compounds **1** [NaCo(HCOO)_3_(H_2_O)]_*n*_ and **2** [NaNi(HCOO)_3_(H_2_O)]_*n*_ present chiral nuclear structures in the *P*2_1_ space group, achieved without adding any chiral induction agent, while compound **5** [KNi(HCOO)_3_]_*n*_ crystallizes in the Sohncke space group *P*6_3_22. We carried out a magneto-structural analysis, including X-ray diffraction, magnetometry, and heat capacity measurements, to comprehend their macroscopic magnetic behavior and evidencing magnetic LRO in all the samples. On this basis, we performed powder and single-crystal neutron diffraction experiments to determine the magnetic structure of the five synthesized CPs.

## Materials and Characterization

All reagents and solvents were used without any further purification

### Synthesis of [NaM(HCOO)_3_(H_2_O)]_*n*_ (M = Co^2+^ or Ni^2+^, compounds **1** and **2**, respectively)

A 5 mL methanolic solution of 0.625 mmol of CoCl_2_·6H_2_O (0.148g) or NiCl_2_·6H_2_O (0.148 g) was added dropwise to a solution of 1.30 mmol of NaSCN (0.105 g) in methanol at constant stirring. After 5 min, this solution was immediately added dropwise to a 10 mL methanolic buffer of 13.25 mmol of HCOOH (0.500 mL, 98%) and 5 mmol of HCOONa (0.340 g) and stirred for 5 min. Then, the solution was left undisturbed, and after 2 weeks, deep red crystals were recovered for [NaCo(HCOO)_3_(H_2_O)]_*n*_ (**1**), and after 1 month, light green powder was recovered for [NaNi(HCOO)_3_(H_2_O)]_*n*_ (**2**).

### Synthesis of [KM(HCOO)_3_]_*n*_ (M = Mn^2+^, Co^2+^, or Ni^2+^, compounds **3, 4**, and **5**, Respectively)

For compound **3**, a 5 mL methanolic solution of 0.625 mmol of MnCl_2_·4H_2_O (0.124 g) was added dropwise to a solution of 1.30 mmol of KSCN (0.120 g) in methanol at constant stirring, then filtered, and kept filtrated. Meanwhile, 10 mL of a methanolic buffer of 13.25 mmol of HCOOH (0.500 mL, 98%) and 5 mmol of HCOOK (0.340 g) was stirred for 5 min and poured in a 20 mL glass test tube. Then, about 1 mL of MeOH was smoothly added to the surface of the buffer, and later, the MnCl_2_/KSCN filtered solution was added dropwise to the tube, aiming for a liquid–liquid diffusion system.

Compounds **4** and **5** were prepared when 5 mL of methanolic solution of 0.625 mmol of CoCl_2_·6H_2_O (0.148 g) or NiCl_2_·6H_2_O (0.148 g) was added dropwise to a solution of 1.30 mmol of KSCN (0.120 g) in methanol at constant stirring. After 5 min, this solution was immediately added dropwise to a 10 mL methanolic buffer of 13.25 mmol of HCOOH (0.500 mL, 98%) and HCOOK (0.340 g) and stirred for 5 min. Then, the solution was left undisturbed; after 1 week, the light pink powder was recovered for [KCo(HCOO)_3_]_*n*_ (**4**), and after 3 weeks, light green crystals were recovered for [KNi(HCOO)_3_]_*n*_ (**5**).

A single crystal of **1, 3**, **4,** and **5** was directly picked up from the reaction media and glued on a glass capillary using epoxy resin. X-ray diffraction data were collected at room temperature on a BRUKER APEX II diffractometer and processed with the APEX3 program suite, using Mo–K_α_ as the X-ray wavelength. Frame integration and data reduction were carried out with the program SAINT, and SADABS was employed for multiscan-type absorption corrections. Using the Olex2^46^ package, the structures were solved with the ShelXT^47^ structure solution program using Dual Space Methods and refined with the ShelXL^48^ refinement package, using least-squares minimization based on *F*^2^. Crystallographic data details on data collection and refinement parameters of the crystal structure are summarized in Table 1. Structure drawings have been elaborated with Crystal Impact’s Diamond 4 software.^49^ Additional data concerning the crystals and the refinement parameters are detailed in the Supporting Information.

**Table 1:** Crystallographic Data for Compounds **1–5**

*Compound*	**1**	**2**	**3**	**4**	**5**
formula	[NaCo(HCOO)_3_(H_2_O)_2_]_*n*_	[NaNi(HCOO)_3_(H_2_O)_2_]_*n*_	[KMn(HCOO)_3_]_*n*_	[KCo(HCOO)_3_]_*n*_	[KNi(HCOO)_3_]_*n*_
*M*_*w*_ [g mol^–1^]	252.99	252.77	229.09	233.09	232.85
space group	*P*2_1_	*P*2_1_	*C*2/*c*	*C*2/*c*	*P*6_3_22
radiation	X-ray MoKα	2.45 Å neutrons	X-ray MoKα	X-ray MoKα	X-ray MoKα
*T* [K]	296	40	296	296	296
*a* [Å]	7.3872(3)	7.3312(4)	10.8160(2)	10.7158(12)	7.0144(4)
*b* [Å]	7.3982(3)	7.3138(10)	9.0584(17)	8.957(1)	7.0144(4)
*c* [Å]	7.9147(4)	7.8155(5)	7.0808(13)	6.8711(8)	8.2698 (4)
β [°]	116.680(1)	116.745(4)	95.175(4)	95.491(3)	90
*V* [Å^3^]	386.50(3)	374.22(8)	690.90(20)	656.47(13)	352.38(4)
*Z*	2	2	4	4	2
ρ [g cm^–3^]	2.010	2.083	2.203	2.358	2.195
μ [mm^–1^]	2.272	4.900	2.490	3.224	3.321
*F*(000)	234	69	452	460	232
sinΘ/λ	0.0708–0.7188	0.0107–0.3778	0.0720–0.7213	0.0729–0.7151	0.0823–0.7201
*R*_int_	0.0184	0.0529	0.0239	0.0241	0.0183
*R*_1_	0.0134	0.0810	0.0186	0.0192	0.0238
*wR*_2_	0.0327	0.0927	0.0499	0.0470	0.0670
*GoF*	1.060	3.400	1.109	1.097	1.194

Magnetic measurements were performed in polycrystalline samples using a Quantum Design Dynacool Physical Properties Measurement System (PPMS) equipped with a Vibrating Sample Magnetometer (VSM). The *dc-*susceptibility data were collected under an applied magnetic field of 1 kOe in the 1.8–300 K temperature range, except for compound **5,** which was measured with 10 kOe, because its magnetic signal was much lower. The isothermal magnetization measurements were performed for **1** and **2** at 2 K, sweeping the applied magnetic field from 0 to ± 90 kOe. Pascal’s constants were considered for diamagnetic corrections.^50^

Heat capacity measurements were performed using a PPMS equipped with a previously calibrated puck calorimeter at zero applied magnetic field in the temperature range of 1.8 to 35 K. The powder sample was pressed to form a pellet, and then a very small amount of grease (Appiezon N) was used to make proper thermal contact between the pellet and a sample platform. Sample response was obtained from the subtraction of the grease signal from the total contribution of heat capacity inside the calorimeter.

A single-crystal neutron diffraction experiment was performed on a 2 × 1 x 1 mm^3^ crystal of compound **1** mounted on a vanadium pin on a four-circle D19 diffractometer at the Institute Laue Langevin (ILL), Grenoble (France). The experiment was carried out using a constant wavelength of 1.45 Å and a closed-circuit displex cooling device. Full data acquisitions were made at 4 and 20 K and were used for the nuclear and magnetic refinements. Unit cell determinations were performed using PFIND and DIRAX programs, and processing of the raw data was applied using RETREAT, RAFD19, and Int3D programs.^51−53^ The data were corrected for the absorption of the low-temperature device using the D19ABSCAN program.^54^Table S1 contains crystallographic data for the refinement of compound **1** at 20 K.

Powder Neutron diffraction experiments were performed on different instruments at several neutron sources: (i) the cold-neutron diffractometer DMC with a wavelength of 2.45 Å^55^ and the thermal high-resolution diffractometer HRPT working with 1.49 Å,^56^ both at the SINQ at the Paul Scherrer Institute (PSI); (ii) the time-of-flight diffractometer POWGEN at the Spallation Neutron Source (SNS) from Oak Ridge National Laboratory (ORNL);^57^ and (iii) the thermal high-intensity two-axis diffractometer D20 at the ILL^58^ in Grenoble, (France), using a wavelength of 1.88 Å. Here, we will show only data collected at DMC, for compounds **2** and **5**, POWGEN for compound **3,** and D20 for compound **4**. Table S2 contains crystallographic data for compounds **2**–**5**.

In all of the powder neutron diffraction experiments, the samples were contained in a 6 mm cylindrical vanadium can and placed inside an Orange cryostat. Patterns were acquired above and below the magnetic order temperature previously determined by *dc*-susceptibility and heat capacity measurements to obtain the structure of both nuclear and magnetic phases for the studied compounds. The data analysis was carried out by performing Rietveld refinement using FULLPROF^59^ code and the WINPLOTR^60^ software. We employed the representational analysis proposed by Bertaut^61^ to determine and label all the possible magnetic structures compatible with their respective space groups and propagation vectors using BasIreps code, included in the Fullprof suite. We also employed the k-SUBGROUPSMAG program from the Bilbao Crystallographic Server (BCS)^62−65^ to determine the magnetic space groups.

## Results and Discussion

### Structural Description

[NaM(HCOO)_3_(H_2_O)]_*n*_, M = Co^2+^ (**1**) or Ni^2+^ (**2**). **1** and **2** are isostructural crystallizing in a monoclinic unit cell with the *P*2_1_ Sohncke space group. The crystallographic parameters are given in Table 1. The asymmetric unit comprises an M^2+^ cation, three formate anions, a sodium ion, and a water molecule. The transition metal cation, M^2+^, is surrounded by six formate anions reaching a hexacoordination (MO_6_) with mean distances of *d*_mean_(Co–O_for_) = 2.094 (2) Å and *d*_mean_(Ni–O_for_) 2.094 (9) Å. Additionally, we calculated the bond valence sum^66^ (BVS) for Co^2+^ and Ni^2+^ ions obtaining values of 2.03 and 1.89, respectively, very close to what is expected (2). Table S3 summarizes the values for all of the compounds.

Moreover, neutron diffraction experiments for **1** at 20 K reveals that *d*_mean_(Co–O_for_) = 2.079 (24) Å, indicating similarity, respects the RT structural refinement. More details about neutron diffraction experiments shall be discussed in the following section.

**Figure 2:**
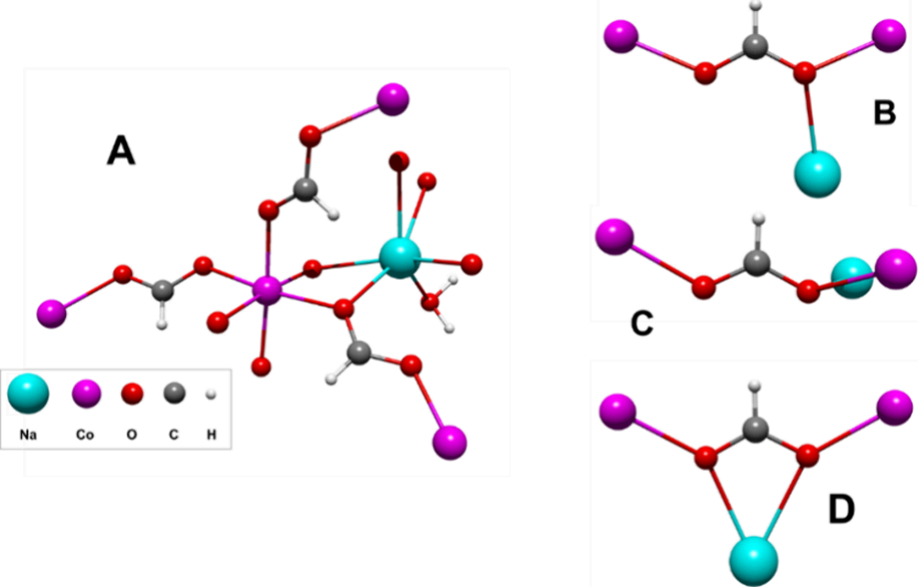
(A) Asymmetric unit of 1. Coordination modes of formate anion in 1 and 2: (B) μ_3_-η^1^:η^2^ “in-plane”; (C) μ_3_-η^1^:η^2^ “out-of-plane” and (D) μ_3_-η^2^:η^2^.

The six formate ligands bridge the M^2+^ cations in an *anti–anti* way with two different coordination modes, giving more complexity to the structure. Thus, considering the M^2+^ and Na^+^ cations, four of the formates present an *anti–anti* bridging (μ_3_–η^1^:η^2^) coordination mode (Figure 2B,C), coordinating one M^2+^ cation by an oxygen atom and one M^2+^ and Na^+^ cation by the second oxygen atom, with *d*(Na–O_for_) = 2.468 (2) Å for **1** and 2.319 (9) Å for **2**. Also, it is worth noticing that two of the formate ligands possess the pair M^2+^/Na^+^ on the formate plane (in-plane, Figure 2B), while in the other two, the pair M^2+^/Na^+^ is located perpendicular to the formate plane (out-of-plane, Figure 2C). These arrangements gave in-plane M–M distances of 5.998 (1) and 5.964 (3) Å for **1** and **2**, respectively, while in the out-of-plane, shorter M–M distances of 5.622 (2) Å for **1** and 5.729 (3) Å for **2**, respectively, were obtained.

The remaining two formate anions present an *anti–anti* chelating–bridging (μ_3_–η^2^:η^2^) coordination mode (Figure 2D), where each oxygen atom coordinates one M^2+^ and Na^+^ cation, obtaining Na–O_for_ bonds in the range of 2.496 (2)–2.596 (2) Å for **1** and 2.477 (12)–2.657 (12) Å for **2** and M–M distances of 5.844 (1) Å for **1** and 5.585 (3) Å for **2**.

**Figure 3:**
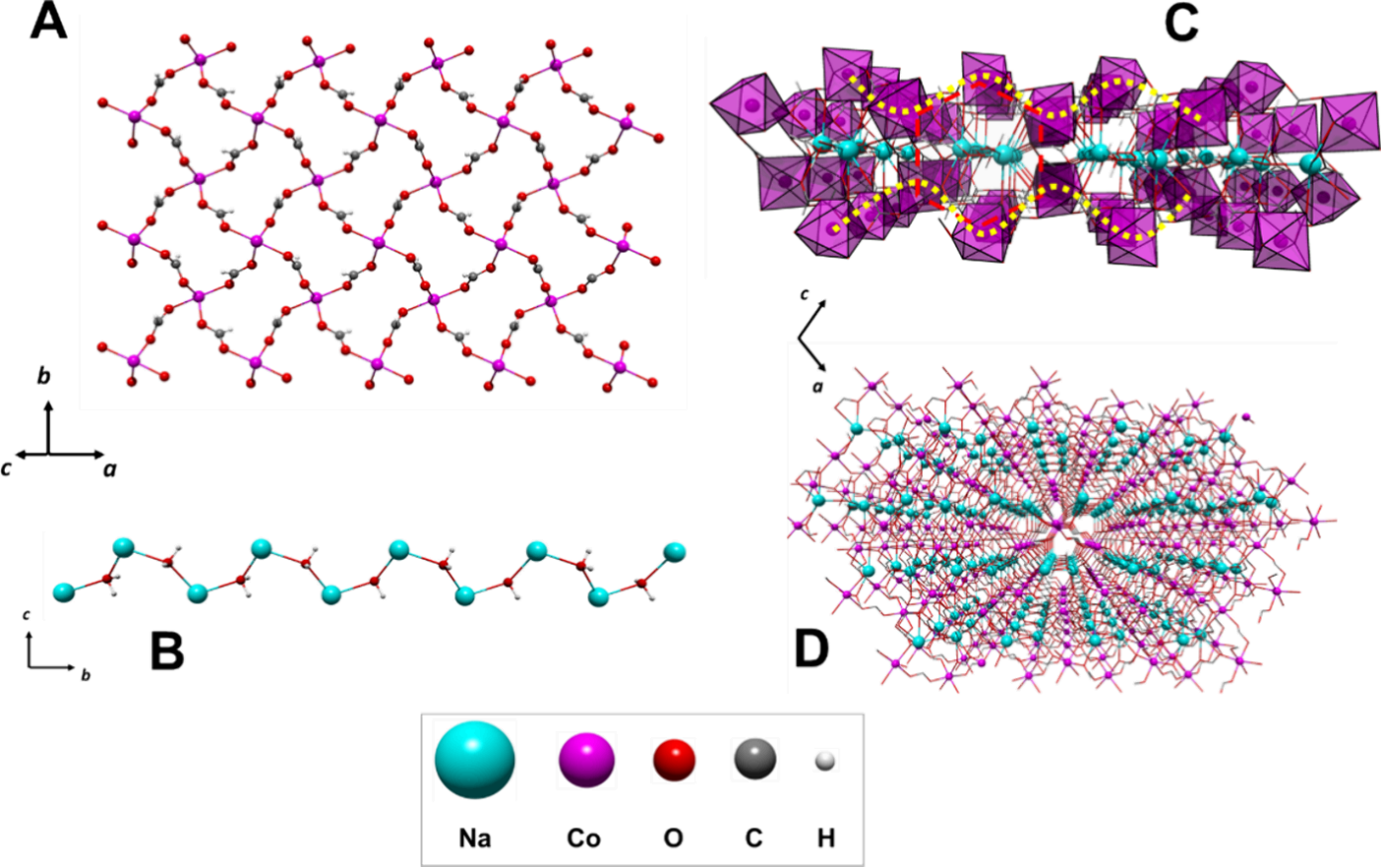
(A) 2D subnet of **1** along the ab plane; (B) sodium–water 1D subnet of **1** viewed from the bc plane; (C) two-layer arrangement of **1** along the *b-axis* perspective, and (D) 3D arrangement for compound **1**.

In **1** and **2**, two subnets are identified considering the M^2+^ or Na^+^ cations. Thus, a 2D substructure composed of M_4_ rhombohedral metal cores is formed by assembling four formate ligands that can be visualized along the ab plane in Figure 3A. The lamellar subnets are stacked between them along the (1 0 1) direction but displaced with respect to the other (Figure 3C). A view along the *c-*axis locates one M^2+^ cation of the one M_4_ core in the center of the rhombohedral arrangement belonging from the above and underneath layers (Figure S1). A second subnet is formed by the Na^+^ cations that are surrounded by three formate anions, interacting by pairs in a monodentate (Figures 2B,C) and chelating mode (Figure 2D), besides two water molecules, giving place to a hexacoordinated environment (NaO_6_), with a high distorted octahedral geometry. Then, a second subnet can be identified considering the Na^+^ cations and the water molecules, arising a zigzag chain that grows along the *b-axis* (Figure 3B), with Na–O_W_ bond lengths in the range of 2.352 (2)–2.520 (2) Å for **1** and 2.167 (11)–2.566 (9) Å for **2**. In this subnet, the Na–O_W_–Na angle is 138.40 (7)° and 138.72 (6)° for **1** and **2**, respectively, while the angle of the O_W_–Na–O_W_ angle is 107.76 (6)° and 115.23 (1)° for **1** and **2**, respectively. Both angles define the zigzag conformation of the Na^+^ chains.

The interaction between the M^2+^ and Na^+^ subnets is a product of the coordination of formate ligands in an *anti–anti* bridging (μ_3_–η^1^:η^2^) and *anti–anti* chelating–bridging (μ_3_–η^2^:η^2^) coordination mode, as described above (Figure 2D), but also by the hydrogen bond interactions between the water molecules and the formate ligands. Interesting to note is that the H_W_–O_for_ distances are 2.088 (22) Å for **1** and 1.857 (9) Å for **2**, in the case of out-of-plane formate ligand, and 1.971 (32) Å for **1** and 1.907 (10) Å for **2**, in the case of in-plane formate ligand. The value of the H_W_–O_for_ noncovalent interactions allows us to infer that the water molecules are relatively fixed in the structure, restricting the thermal fluctuations and then fixing the hydrogen positions (Figure S2). Furthermore, the slight difference between the hydrogen bonds could be related to the in-plane and out-of-plane conformations that acquire the formate ligands in the structures of **1** and **2**.

Also, it is important to remark that the position of the M^2+^ cations on the whole net gives place to a corrugated shape in the layers, where a wave shape has a displacement relative to each other, raising hexagonal cavities between the layers (Figure 3C). Figure 3D shows a complete view of these two new 3D networks, [NaM(HCOO)_3_(H_2_O)]_*n*_, with M = Co^2+^ or Ni^2+^.

[KM(HCOO)_3_]_*n*_ M = Mn^2+^ (**3**) or Co^2+^ (**4**) have been reported previously.^39,67,68^ Both compounds are isostructural, crystallizing in a monoclinic unit cell with the *C*2/*c* space group. The lattice parameters of **3** and **4** are found in Table 1. A brief description of the structures is given to understand the magneto-structural correlation better.

**Figure 4:**
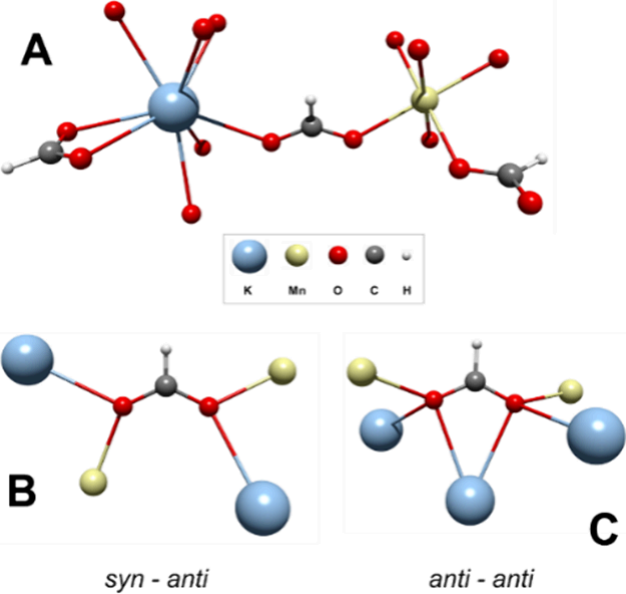
(A) Asymmetric unit of compound **3**, (B) *syn–anti* coordination mode, and (C) *anti–anti* coordination mode for the formate ligand in compounds **3** and **4**.

In **3** and **4**, the cations are coordinated only by formate anions, having an asymmetric unit composed of a hexacoordinated M^2+^ cation (MO_6_), three formate ligands, and a potassium ion. The M^2+^ cation possesses a slightly distorted octahedral environment (Figure 4A), and a *d*_mean_(Mn–O) of 2.177 (1) Å and a *d*_mean_(Co–O) of 2.099 (1) Å for **3** and **4**, respectively. BVSs were calculated, obtaining values of 2.11 for the Mn^2+^ ion in **3** and 1.99 for the Co^2+^ ion **4**, in both cases close to what is expected (**2**) (Table S3).

Considering the M^2+^ and K^+^ cations, the formate ligands are characterized by presenting two bridging coordination modes, *syn–anti* bridging μ_4_–η^2^:η^2^ and *anti–anti chelating–bridging* μ^5^–η^3^:η^3^. For the *syn–anti* μ_4_–η^2^:η^2^, the plane formed by both M–O–K moieties is coplanar with the plane of the formate ligand, with M–O distances in the range of 2.1678 (10) and 2.1762 (10) Å for **3** and 2.095 (1) and 2.101 (1) Å for **4,** while the M–M bond lengths achieved are 5.749 (1) Å and 5.645 (1) Å for **3** and **4**, respectively. On the other hand, in the *anti–anti* conformation, the M–O–K moieties are adopting a perpendicular disposition with respect to the formate ligand plane, with an expected longer M–O bond distance of 2.188 (1) Å for **3** and 2.101 (1) Å for **4**, affording M–M distances of 6.191 (1) Å for **3** and 6.082 (1) Å for **4**.

Similar to compounds **1** and **2**, a two-dimensional subnet based on M_4_ rhombohedral units of the M^2+^ cation and the *syn–anti* formate bridge is visualized on the bc crystal plane, forming a lamellar arrangement. Meanwhile, the potassium ions form a second 1D substructure, which can be understood as a zigzag chain along the *c* crystallographic axis. Herein, the *anti–anti* formate ligand has a linking role between the cations.

**Figure 5:**
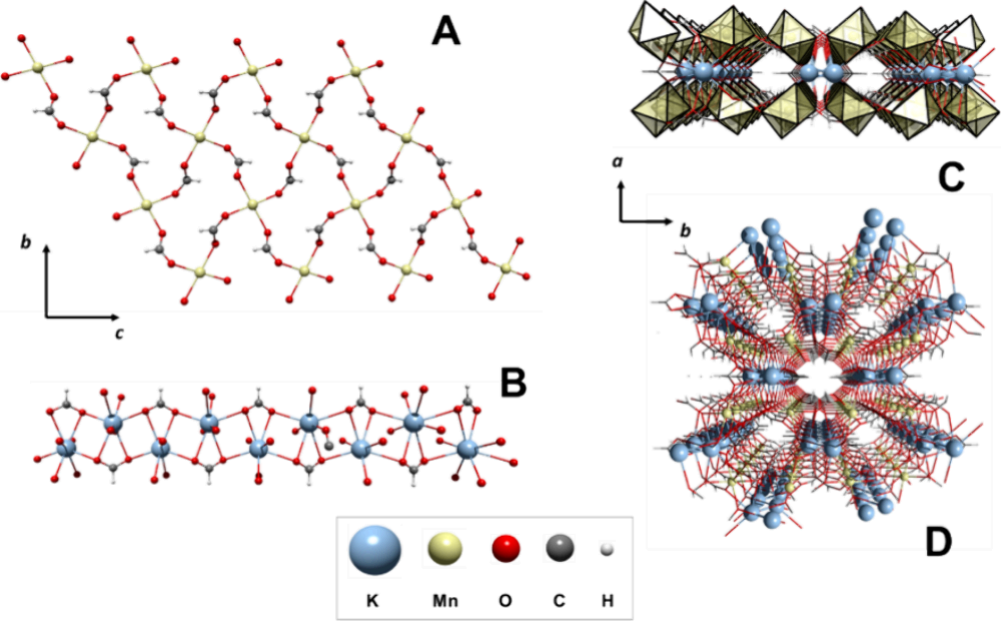
(A) M^2+^/HCOO layers, (B) K^+^/HCOO chains, (C) two-layer arrangement along the *a-axis* perspective, and (D) 3D arrangement for compound **3**.

Therefore, the tridimensional polymer structure arises as the zigzag potassium chains are located between the M^2+^/HCOO layers, arranging tubular cavities that grow along the *c* axis, with a diameter of ∼4.2 Å, where the hydrogen atoms from both types of formate ligands point to the center of it (Figure 5C).

Compound **5,** [KNi(HCOO)_3_]_*n*_, crystallizes in a hexagonal unit cell with a noncentrosymmetric Sohncke space group *P*6_3_22 (Table 1). This unreported CP is isostructural to the {[NH_4_^+^][M(HCOO)_3_]}_*n*_ (M = Mn^2+^, Co^2+^, Ni^2+^) presented in 2007 by Wang et al.^41^ and to the alkali/nickel [ANi(HCOO_3_)]_*n*_ (*A* = Rb^+^, Cs^+^) structures of Bovill and Saines.^40^ The asymmetric unit comprises a hexacoordinated nickel cation, three formate ligands, and a potassium cation. The hexacoordination of the nickel ion is reached by six formate ligands with the same *d*(Ni–O) = 2.064 (3) Å, but the octahedral geometry is evidently distorted due to the O–Ni–O angles. The BVS calculated value for the Ni^2+^ ion is 1.98, in agreement with the expected value (**2**). Potassium ion is also hexacoordinated with a geometry that could be described as a trigonal antiprism with all K–O distances of 2.843 (3) Å (Figure S3A).

For the Ni^2+^ cations, the formate ligands are acting in an *anti–anti* bridging μ_4_-η^2^:η^2^ coordination mode, while the potassium ions are bridged in a *syn–syn* manner (Figure S3B). The four coordinated cations do not share the same plane, where the K–O–Ni triangles formed in each oxygen atom of the ligand possess a dihedral angle between each other of 50.41 (5)° (Figure S3C). Moreover, the formate bridge affords Ni–Ni distances of 5.787 (1) Å, which would be interesting later from the magnetic property perspective.

**Figure 6:**
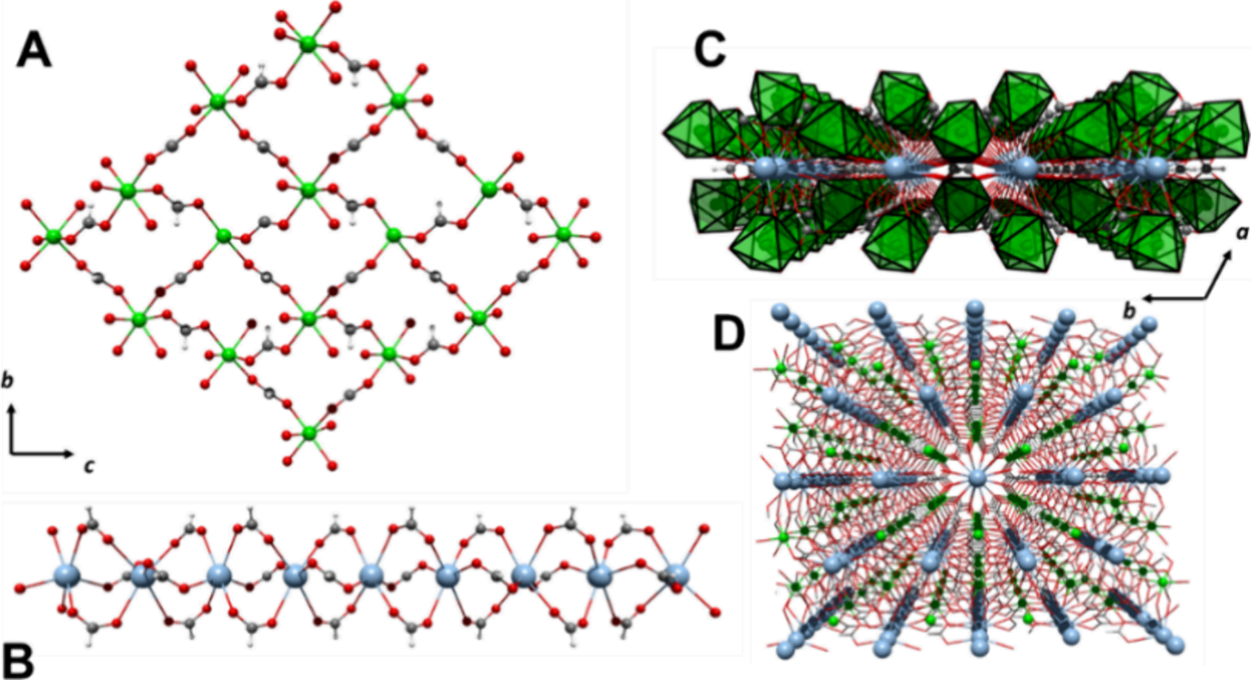
(A) Ni^2+^/HCOO 2D substructure, (B) K^+^/HCOO 1D substructure, (C) 3D arrangement of two Ni^+^/HCOO layers, and (D) General view of the 3D arrangement for compound **5.**

Two subnets arise from the 3D arrangement. On one hand, 2D subnet-based M_4_ units of Ni^2+^/HCOO are identified on the bc crystallographic plane (Figure 6A), where each cation is connected to other four nickel centers by four formate ligands, forming a layer with corrugated shape, similar to compounds **1** and **2**. Meanwhile, 1D substructures composed of potassium ions linked by formate ligands grow along the *c* crystallographic axis, where the cations are disposed linearly (Figure 6B). The layers are connected by a pair of formate anions in the *cis* position, giving rise to stacking ordering along the *b-axis*. The three-dimensional arrangement of the CP is completed when the 1D subnet is identified inside the hexagonal cavities that remain between the Ni^2+^/HCOO layers (Figure 6C,D).

### Magneto-thermal Characterization

**Figure 7:**
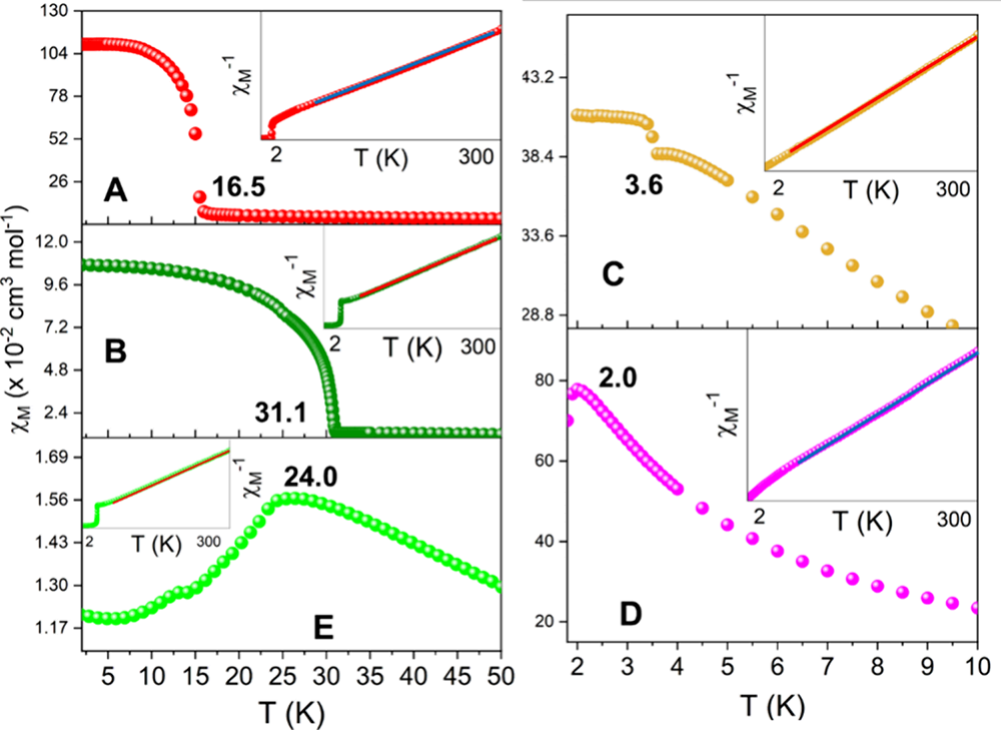
DC magnetic susceptibility versus temperature (main) and fit of the inverse of magnetic susceptibility (insert) and for compounds (A) **1**, (B) **2**, (C) **3**, (D) **4**, and (E) **5.**

**Figure 8:**
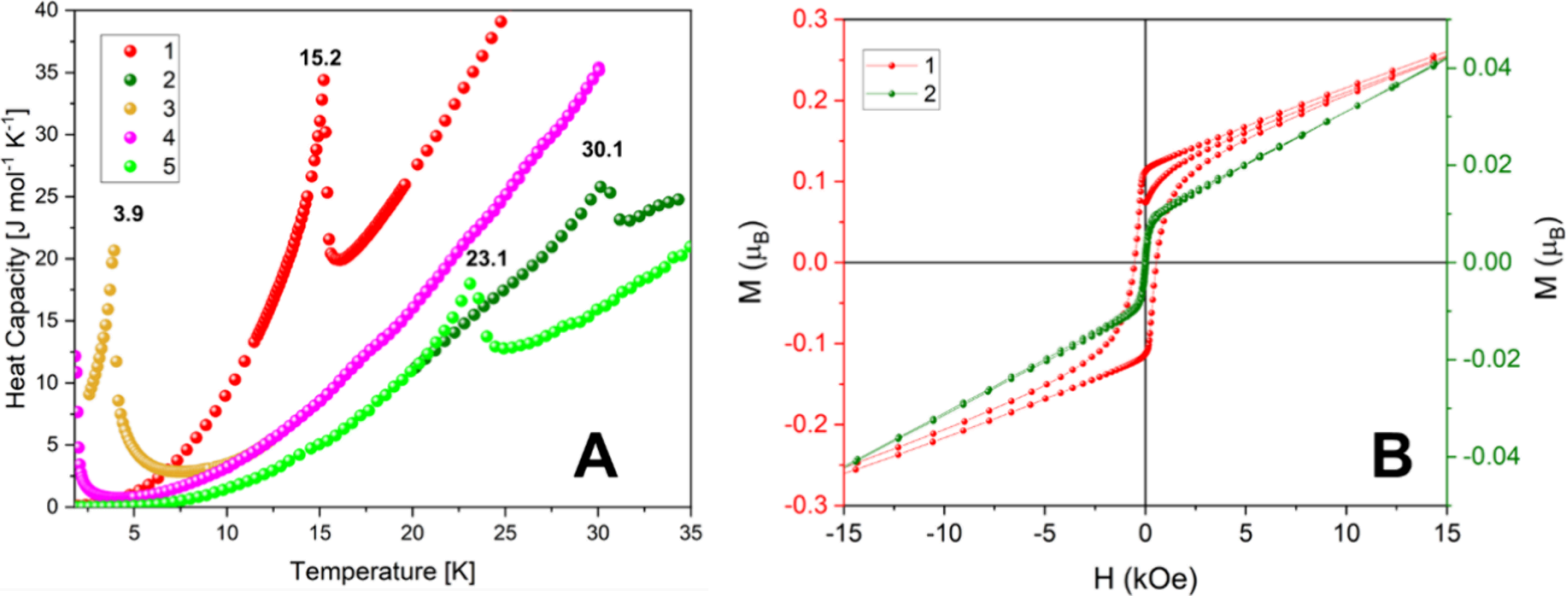
(A) Heat capacity versus temperature measurements for compounds **1**–**5**. Ordering temperatures are written in the peaks. (B) Magnetization versus magnetic field cycles for compounds **1** (red *y*-axis) and **2** (green *y*-axis).

*dc*-Magnetic susceptibility and isothermal magnetization measurements were performed to evaluate the magnetic behavior of compounds **1**, **2**, and **5** and to corroborate the magnetic behavior of compounds **3** and **4**, previously reported. Heat capacity measurements were also performed to elucidate the phase transition in compounds **1**–**5**. Figure 7 shows the magnetic *dc*-magnetic susceptibility (χ_*M*_) curves measured for compounds **1**–**5** as a function of the temperature, in the range of 2 to 50 K. χ_*M*_(*T*) plots in the range of 2–300 K is depicted on Figure S4. The χ_*M*_(*T*) for compound **1** shows a Curie–Weiss behavior from 300 to ∼50 K (see insert Figure 7A). At 16.5 K, χ_*M*_ shows an abrupt rise from 6.26 × 10^–2^ to 1.09 cm^3^ mol^–1^, leaving this value relatively constant to 2 K (Figure 7A), becoming more prominent by using magnetic fields of less intensity (Figure S5). This abrupt rise of χ_*M*_ agrees with the peak observed at *T_N_* = 15.2 K at the heat capacity versus temperature curve, indicating the presence of magnetic LRO, shown in Figure 8A. The value of the asymptotic Curie temperature (θ_CW_ = −47.5 (7) K) extracted from the fit of χ_*M*_^–1^ vs *T* curve indicates the presence of antiferromagnetic (AF) interactions on average of *zJ*_**1**_**/***k*_B_= −38.0 (6) K, (*zJ/k*_B_ = 3θ_CW_/*S*(*S*+1)). The experimental fitted Curie constant (3.43 (1) cm^3^ K mol^–1^) for **1** is higher than the expected for isotropic ions with *S* = 3/2 and *g* = 2, indicating the anisotropic nature of the Co^2+^ cations. The values of the fitted parameters are listed in Table 2. The theoretical values of the Curie constant are listed in Table 2 under two different approaches: (i) spin only approach in which the effective magnetic moment is read as  with *g* = 2 and (ii) weak spin orbit coupling approximation, in which orbital and spin angular moments are considered independently. Under this approach, some authors define the effective magnetic moment as . Considering a  and allowing to vary the Landé *g*-factor in the fit, we arrive for compound **1** to the value 2.71 (1), also listed in Table 2.

**Table 2:** Magnetic Parameters of **1**–**5**, Obtained from the Fit of the χ_*M*_^–1^(T) Curves

compound	**1**	**2**	**3**	**4**	**5**
*C*_exp_ [cm^3^ K mol^–1^]	3.43(1)	1.37(1)	4.41(1)	3.65(1)	1.23(1)
*C*_theo-S_ [cm^3^ K mol^–1^]	1.875	1.00	4.375	1.875	1.00
*C*_theo-SO_ [cm^3^ K mol^–1^]	3.38	2.49	4.38	3.38	2.49
*g-*factor	2.71(1)	2.34(1)	2.01(1)	2.79(1)	2.22(1)
*T*_*N*_ [K]	15.2	30.1	3.9	∼2.0	23.1
θ_CW_ [K]	–47.5(7)	–63.3(6)	–3.45(40)	–14.4(4)	–43.1(2)
*zJ*/*k*_B_ [K]	–38.0(6)	–94.9(1)	–1.18(2)	–11.5(4)	–64.7(3)

Isothermal magnetization versus magnetic field cycles at 2 K for compound **1** was also measured up to 90 kOe. They are depicted in Figure 8B in the range of −15 to 15 kOe. The curve abruptly increases at low fields and then, after decreasing the slope, continues monotonically increasing until 90 kOe. Some small hysteresis of 540 Oe was detected with a remnant magnetization of *M*_*r*_*ca*. 0.11 μ_B_, enlightening the presence of a weak ferromagnetic component.

The shape of the χ_*M*_ at low temperatures, together with the hysteresis plots, *M*(*H*), could be understood with the presence of some weak ferromagnetic component coming from a slightly canted AF LRO of the Co^2+^ cations. An estimation of the canting angle (α) for **1** considering the remnant magnetization *M*_*r*_ and the value of the effective magnetic moment extracted from the Curie constant gives a value of α *ca*. 3.4°.

A similar behavior is observed for compound **2**; however, the increment of χ_*M*_ is observed around *ca*. 31 K, going from 1.26 × 10^–2^ to 0.107 cm^3^ mol^–1^, and as was observed for **1**, it reaches higher values as the intensity of the magnetic field decreases (Figure S6). The heat capacity measurement shows a peak at *T*_*N*_ = 30.1 K (Figure 8A) that is in agreement with the χ_*M*_ curves. The fit of χ_*M*_^–1^(T) for compound **2** gave *C*_exp_ and θ_CW_ values of 1.37 (1) cm^3^ K mol^–1^ and −63.3 (6) K, respectively. It conduces to a *g* value of 2.34 (1) K for the Ni^2+^ and an AF average *zJ*_**2**_**/***k*_B_ = −94.9 (1) K (using the same approximation as that for **1**). Also, very small hysteresis (∼25 Oe) and remanence (*M*_*r*_ ∼1 × 10^–2^ μ_B_) are detected for **2** with values one order of magnitude smaller than in **1**. For compound **2,** a canting angle of 0.5° is estimated.

Susceptibility versus temperature for compound **3** indicates a predominant paramagnetic behavior from room temperature down to *ca*. 5 K. Then, it abruptly rises at 3.6 K, in agreement with previous reports,^67^ reaching a maximum value of 0.409 cm^3^ mol^–1^ (Figure 7C), which remains constant to 1.8 K. Similar to compounds **1** and **2**, this abrupt increase represents a weak ferromagnetic behavior, studied through a hysteresis loop (Figure S7), obtaining an H_C_ ∼100 Oe and a magnetization remanence of *M*_*r*_*∼*0.006 μ_B_*,* being difficult to estimate a canting angle with this very small value. Moreover, the anomaly in the χ_*M*_ curve could indicate some magnetic LRO in agreement with the anomaly at *T*_*N*_ = 3.9 K observed in the heat capacity versus temperature curves (Figure 8A) for compound **3**. The susceptibility curve shows a small hump (just before *T_N_*) possibly related to a crossover from low-dimensional (1D/2D) to 3D magnetic ordering. The fit of χ_*M*_^–1^(*T*) for compound **3** gives *C*_exp_ and θ_CW_, respectively, 4.41 (1) cm^3^ K mol^–1^ and −3.45 (40) K, from a *g* value of ∼2.01 for the Mn^2+^ and an AF average of *zJ***_3_**/*k*_B_ = −1.18 (2) K.

For compound **4**, the magnetic susceptibility versus temperature plot shows a paramagnetic behavior from room temperature down to 2 K, where a maximum value of 0.776 cm^3^ mol^–1^ is observed at 2.0 K (Figure 7D), indicating that an AF LRO could probably appear below 2 K. The heat capacity curve for **4** suggests that a peak is developing for temperatures close to *T*_N_*∼*2 K, although it is not clearly visible because the lowest limit of the experimental temperature is 1.8 K. The analysis of the χ_*M*_^–1^(T) measurement at high temperature gives values for *C*_exp_, θ_CW_, and *zJ***_4_**/*k*_B_ of 3.65 (1) cm^3^ K mol^–1^, −14.4 (4) K, and −11.5 (4) K, respectively. Also, the difference between *C*_exp_ and *C*_theo−S_ for **4** could be understood by the anisotropy of the Co^2+^ cation, and it agrees with the value obtained for **1** (*g*-factor = 2.79 (1) for **4**). The values obtained for *C*_exp_ and *M*_sat_ (∼2.5 μ_B_ at 2 K, Figure S8A) agree with the work of Duan et al.;^39^ however, they found a larger theta value (−24.8 K), which is almost double that of our observation.

The magnetic susceptibility as a function of temperature for compound **5** presents a Curie–Weiss behavior from room temperature until *ca*. 24 K, where a peak is reached with a value of 1.56 × 10^–2^ emu mol^–1^ (Figure 7E). This temperature also coincides with the temperature at which a peak in the heat capacity versus temperature is observed for **5** (Figure 8A), indicating a magnetic LRO of AF nature. Curie–Weiss fitting on the inverse susceptibility plot drops *C*_exp_ = 1.23 (1) cm^3^ K mol^–1^, higher than the expected value (1.00 cm^3^ K mol^–1^) but agrees with the behavior of *C*_exp_ of nickel-based compound **2**, acquiring an estimated g-factor of 2.22 (1). Additionally, the same analysis affords a θ_CW_ = −43.1 (2) K and *zJ*_5_/*k*_B_ = −64.7 (3) K, confirming the AF nature of the exchange between the nickel ions on **5**, being both values very close to the obtained for **1**, observing that a similar behavior is present in both Ni^2+^ CPs. In Table 2 are listed the parameters obtained from the analysis of the χ_M_^–1^(*T*) curves at high temperatures. Isothermal magnetization measurement at 2 K shows no saturation with *M*_max_ values lower than the expected for a Ni^2+^ (2.0 μ_B_), in agreement with strong AFM coupling between the ions (Figure S8B).

Moreover, a difference in the lattice heat capacity is observed between compounds **1** and others. We do not have a clear explanation at this moment, but we attribute it to the presence of water molecules in the structure because, in fact, for compound **1**, after relatively short times in air exposition, some dehydration transformations are observed, not present in other compounds.

### Neutron Diffraction Analysis

Neutron diffraction measurements were performed to investigate the magnetic structure of compounds **1**–**5**. Compound **1** was measured on a single-crystal D19 diffractometer at the ILL using a wavelength of 1.45 Å at 20 K (paramagnetic phase) and at 4 K (ordered phase). At the lowest temperature, the increase of intensity detected in three (0 *k* 0) reflections with odd *k* is indicative of some magnetic LRO with a propagation vector **k** = (0 0 0). By using representational analysis (with the help of BasIreps), we decomposed the magnetic representation Γ_M_, for the Co^2+^ cation located at the 2*a* Wyckoff position of the space group *P*2_1_, as a direct sum of the irreducible representations (*irreps*) mΓ_1_ and mΓ_2_ of the little group of **k** (*G***_k_** = *P*2_1_) as shown in Table 3. Both *irreps* mΓ_1_ and mΓ_2_ are real and one-dimensional.

**Table 3:**
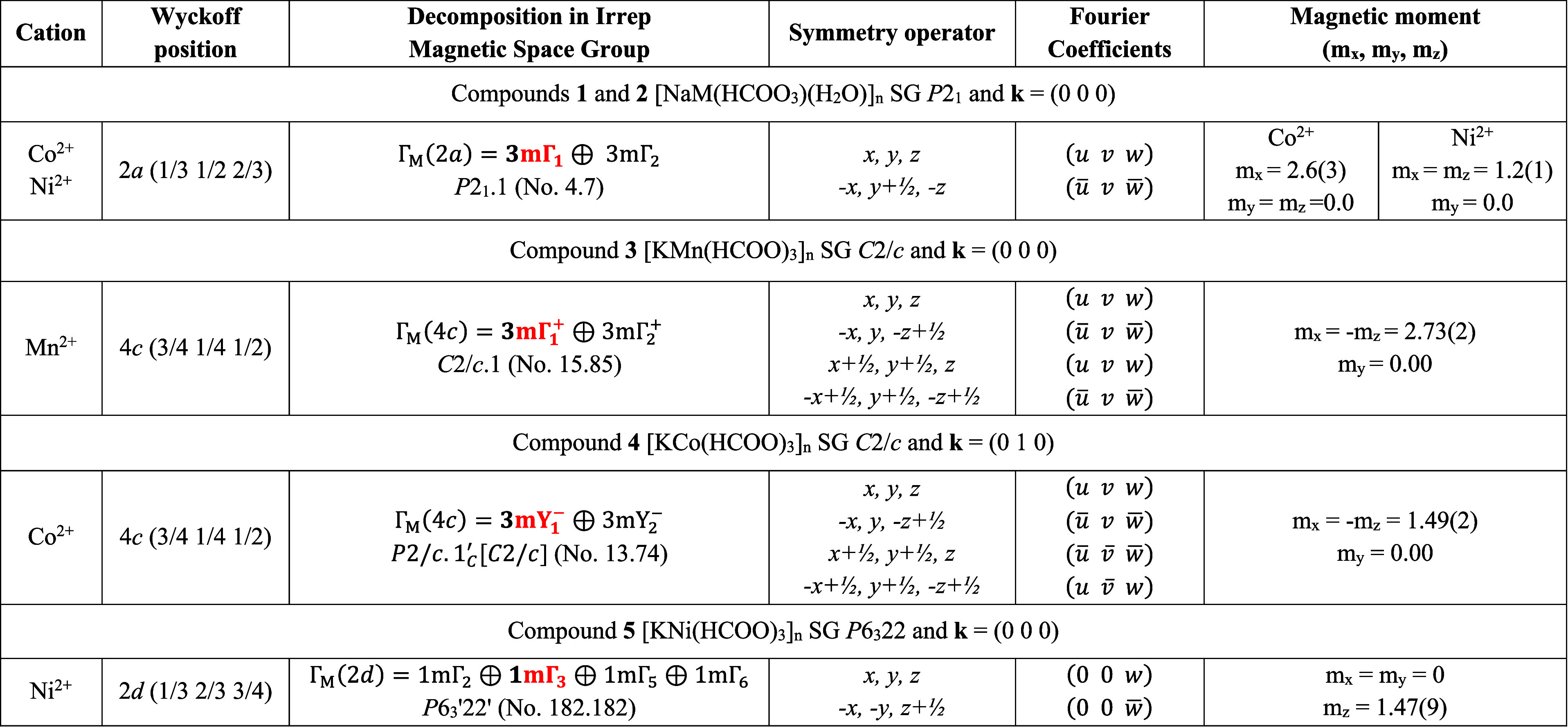
Irreducible Representations Decomposition, Fourier Coefficients, Site Symmetry, Magnetic Space Groups, and Fitted Magnetic Moment Components for Cation Sites in Compounds **1**–**5**, which Give the Best Agreement for Rietveld Refinement in the Low-Temperature Patterns^a^

a In red, the *irreps* that fits the data.

A simple calculation of the magnetic structure factor (**M**_**q**_) and the magnetic interaction vector (**M**_⊥**q**_) for the (0 *k* 0) lines with *k*-odd for the space group *P*2_1_ shows that for mΓ_2_ (Table S4), the magnetic interaction vector is null **M**_⊥(0*k*0)_ = 0, and therefore, any magnetic structure described with mΓ_2_ will never explain the magnetic contribution experimentally observed in (0 1̅ 0), (0 3̅ 0), and (0 5̅ 0), in the ordered phase of **1**. Consequently, the only possibility points to a magnetic structure labeled with mΓ_1_ that allows an AF component perpendicular to the *b*-axis and a ferromagnetic component parallel to the *b*-axis. In our neutron diffraction data, we detect only the AF component with a value of 2.63 (35) μ_B_. The ferromagnetic component along *b* will never be observed in (0 *k* 0), and within the precision of our experiment, it is indetectable in other lines. However, symmetry allows it, and therefore, it could explain the weak ferromagnetic signal observed at the magnetic susceptibility curve measured for **1**. On the other hand, the magnetic moment fitted is comparable to the 3 μ_B_ expected for a *S* = 3/2 as Co^2+^ with *g* = 2. Figure S9 contains a plot of the Rietveld refinement of the data.

Additionally, using the k-SUBGROUPSMAG tool from the BCS, we determined the possible magnetic subgroups associated with the parent group *P*2_1_ and compatible with **k** = (0 0 0). In this sense, the magnetic subgroup consistent with the *irrep* mΓ_1_ is *P*2_1_.1 (No. 4.7).

**Figure 9:**
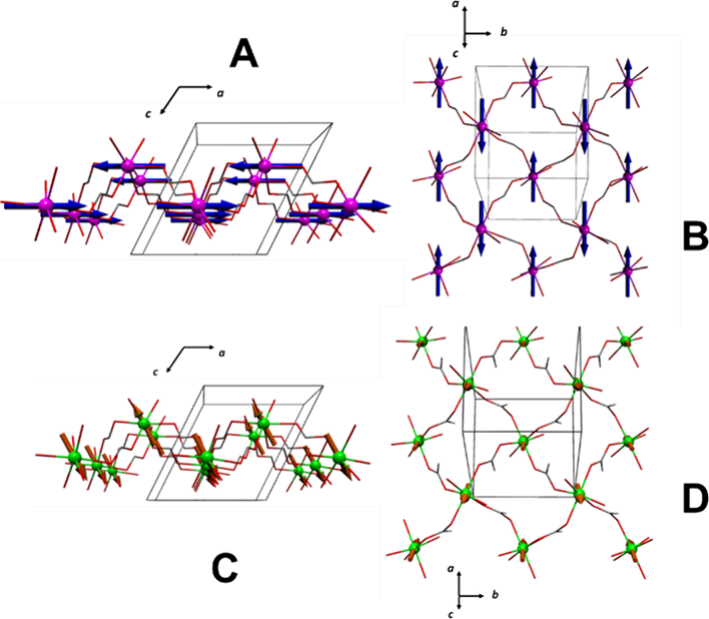
Magnetic structures for compounds **1** (A,B) and **2** (C,D).

The magnetic structure can be described as AF corrugated layers in the ab plane, detailed previously and depicted in Figures 3A and 9B, in which the magnetic moment points toward the *a*-axis, coupled antiferromagnetically along the *c-axis* (Figure 9B). This is consistent with the magnetic coupling expected for the *anti–anti* bridges inside the corrugated lamellar architectures. Furthermore, considering that formate bridges connect the corrugated layers in an *anti–anti* manner, an AF interaction should also be expected between the layers.

**Figure 10:**
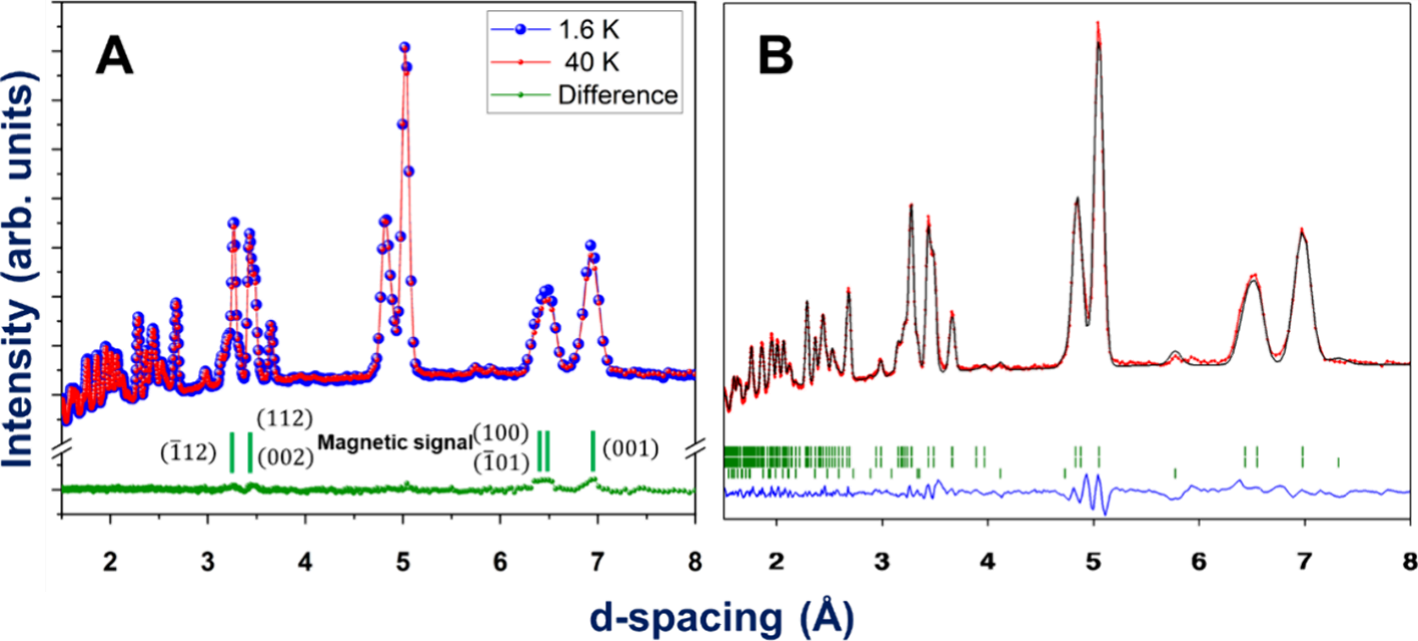
For compound **2**.(A) Diffractograms measured at 40 K (red), 1.6 K (blue), and difference (green). The magnetic signal is indexed with **k** = (0 0 0). (**B**) Rietveld refinement of the 1.6 K diffractogram. The experimental points are in red, the fit is the black continuous line, and the difference between data and fit is the blue line. The green lines signal the positions of the Bragg lines for the nuclear and magnetic phases. The lower green lines correspond to the impurity of [NaNi(HCOO)_3_]_*n*_.

Compound **2** was measured at the DMC instrument at the SINQ facility of the Paul Scherrer Institute, using a constant wavelength of 2.45 Å for the measurements at 40 and 1.6 K. A slight increase in the diffraction peaks (1 0 0) and (1̅ 0 1) around 6.5 Å and (0 0 1) at 7 Å in the *d*-spacing in the low-temperature pattern is indicative of some magnetic LRO with propagation vector **k** = (0 0 0). This can be observed in Figure 10A. As both compounds **2** and **1** are isostructural, we can use the same decomposition of Γ_M_ obtained previously for **1**.

The Rietveld refinement of the pattern in the ordered phase of compound **2** shows a better agreement using mΓ_1_ (Figure 10B), which describes an AF ordering between the nickel ions along the ac plane with a ferromagnetic component along *b* (see Table 4). The fitting reveals magnetic moment components of m_*y*_ = 0 and m_*x*_ = m_*z*_ = 1.25 (10) with a total magnetic moment of 1.8 (2) μ_B_, close to the 2 μ_B_ expected for this cation. Like **1**, the magnetic space group for **2** is *P*2_1_.1 (No. 4.7). A neglectable impurity, impossible to be refined, possibly [NaNi(HCOO)_3_]_*n*_, indexed with the space group *P*6_3_22 (*a* = 6.6673 Å *c* = 8.237 Å), is detected in the powders of compound **2** (see Figure 10B). Like its cobalt isomorph, **1**, this 2D AF corrugated structure is coupled antiferromagnetically along the *c* axis, probably also due to the *anti–anti* way of the formate bridge that connects the layers in **2**. In Figure 9C,D, the magnetic structure of **2** is schematized.

**Figure 11:**
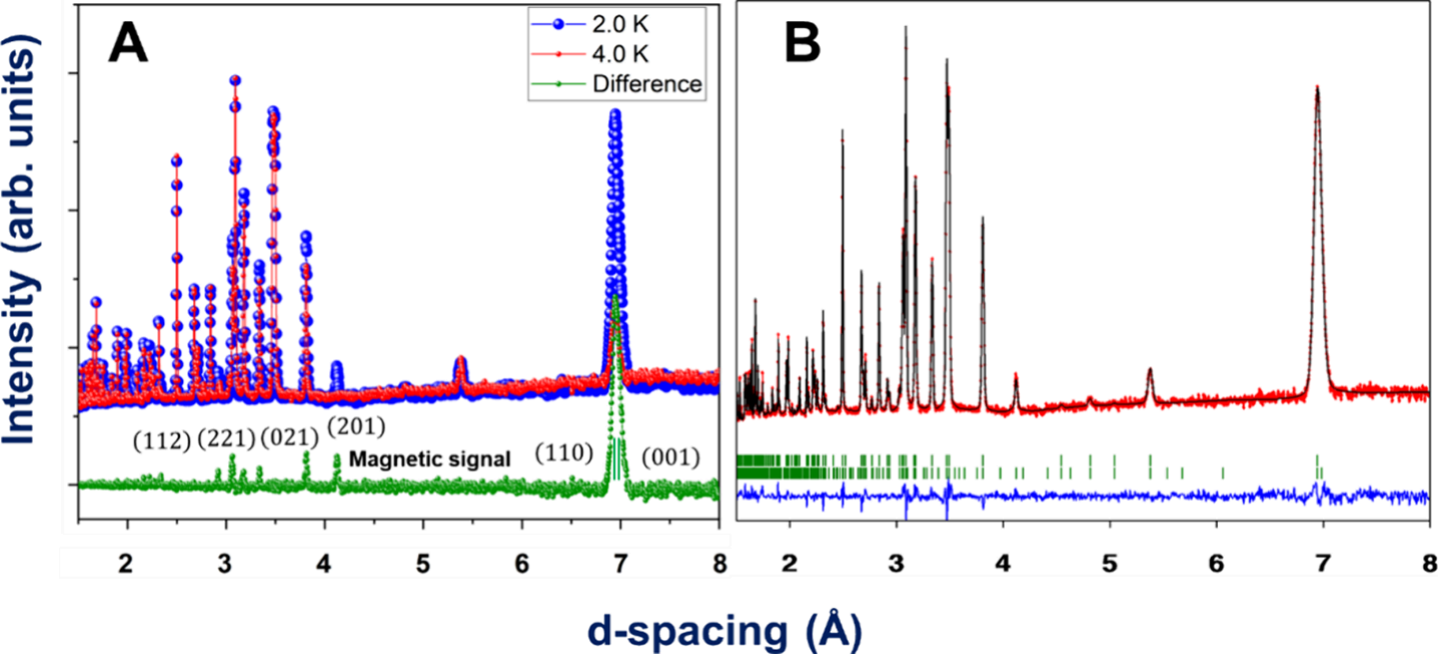
For compound **3.** (A) Diffractograms measured at 4 K (red) and 2 K (blue) and differences (green). The magnetic signal is indexed with **k** = (0 0 0). (B) Rietveld refinement of the 2 K diffractogram. The experimental points are in red, the fit is the black continuous line, and the difference between data and fit is the blue line. The green lines are signaling the positions of the Bragg lines for the nuclear and magnetic phases.

Compound **3** was measured at 4 K in the paramagnetic phase and at 2 K in the ordered phase in the time-of-flight instrument POWGEN (see Figure 11A). In the low-temperature pattern, several nuclear Braggs peaks increase their intensity, e.g., (1 1 0), (0 0 1), (2 0 1), and (0 2 1), which is indicative of magnetic LRO indexed with a propagation vector **k** = (0 0 0) and in agreement with the susceptibility and heat capacity measurements. The magnetic representation for Mn^2+^ at the 4*c* Wyckoff position of the *C*2/*c* space group can be decomposed as a direct sum of two *irreps* (see Table 3 and Table S5). For compound **3**, the Rietveld analysis of the ordered phase pattern has better agreement with the symmetry described by mΓ_1_^+^, which allows an AF ordering with magnetic moments contained in the ac plane, and ferromagnetism along the *b* axis (see Table 3), which allows the canting along the *y*-axis, similar to compounds **1** and **2**. This is in agreement with the stair-step behavior of the magnetic susceptibility values near the ordering temperature. The magnetic space group *C*2/*c* (No. 15.85) describes the symmetry labeled by mΓ_1_^+^.

**Figure 12:**
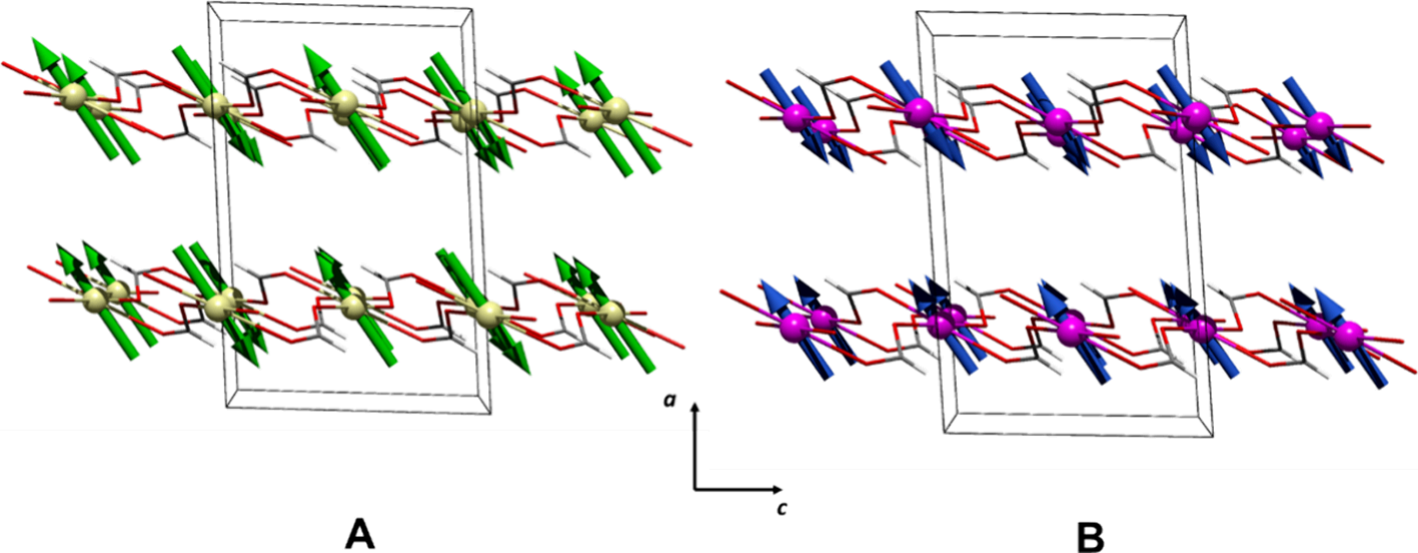
Magnetic structure of compounds (A) **3** and (B) **4**.

The best fit of the new magnetic contribution is reached with m_*x*_ = −m_*z*_ = 2.73 (2) and m_*y*_ = 0. It gives an averaged magnetic moment for manganese ions of 3.9 (3) μ_B_, which is lower than the expected value for a *S* = 5/2 ion (5 μ_B_). This can be due to the fact that measurements are done at temperature, *T* = 2 K, close to its Néel temperature, *T*_N_ = 3.9 K, and therefore, the magnetic moments could be still strongly affected by the thermal fluctuations. For compound **3**, the magnetic structure can be described by layers along the bc plane in which the magnetic moments are ordered antiferromagnetically along the (1 0 1) direction. These layers are coupled ferromagnetically along the *a-*axis (see Figure 12A and Figure S10). The magnetic moment has components only along the *x* and *z* axes, affording an AF ordering in the ac plane of the system, specifically in the Mn/μ_4_-HCOO layers described previously, allowing us to infer that the *syn–anti* formate bridges play an important role in the magnetic interactions of the system.

**Figure 13:**
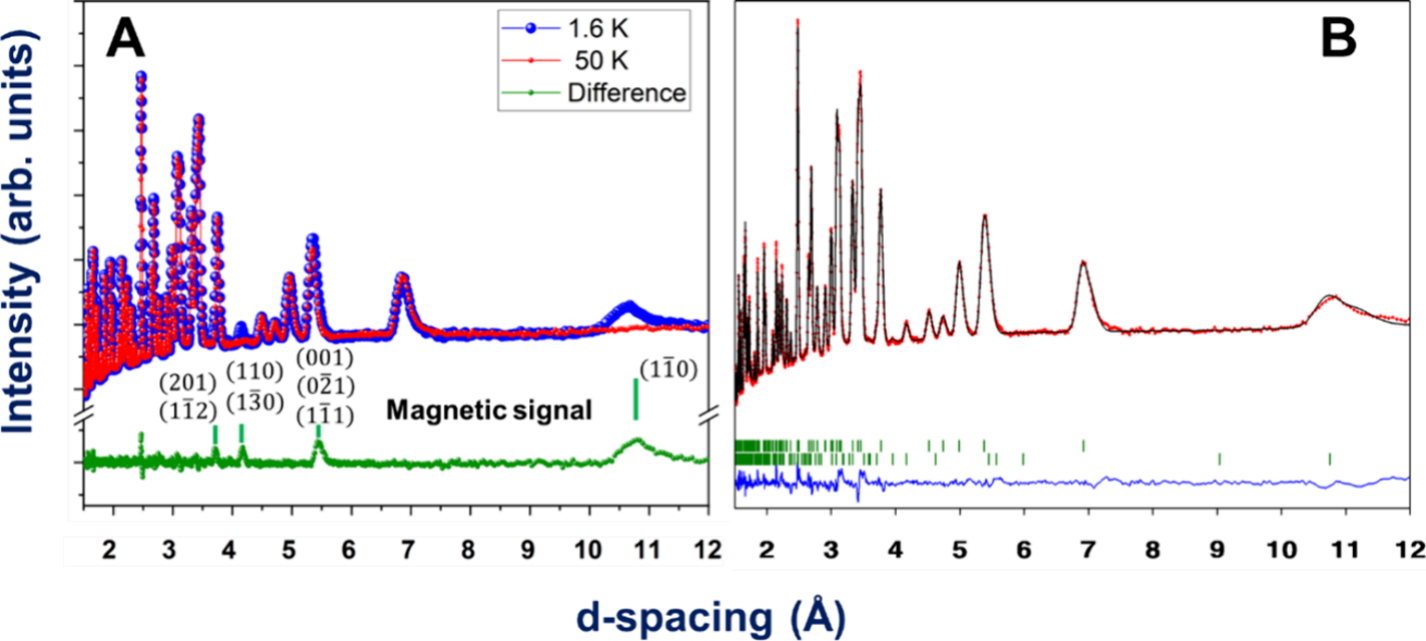
For compound **4**.(A) Diffractograms measured at 50 K (red) and 1.6 K (blue) and differences (green). The magnetic signal is indexed with **k** = (0 1 0). (**B**) Rietveld refinement for the 1.6 K diffractogram. The experimental points are in red, the fit is the black continuous line, and the difference between data and fit is the blue line. The green lines are signaling the positions of the Bragg lines.

Compound **4** was measured with λ = 1.88 Å at the D20 instrument of the ILL. Diffractograms were collected at the paramagnetic phase (50 K) and the ordered phase (1.6 K), as determined from heat capacity measurements. In the low-temperature pattern, the appearance of new peaks and the increase of intensity in other indexed Bragg’s reflections (Figure 13A) indicate magnetic LRO. The new magnetic signal is indexed with a propagation vector **k** = (0 1 0), indicating that the new magnetic symmetry has lost the centering translation (1/2 1/2 0) of the parent group *C*2/*c*. The Co^2+^ cations are located at the Wyckoff position (4*c*) of the *C*2/*c* space group, and the propagation vector is **k** = (0 1 0). The magnetic representation can be decomposed for these constraints as described in Table 3 and Table S6. The fit of the magnetic contribution for compound **4** has better agreement using mY_1_^–^, different from the representation used for the isomorphous compound **3** (mΓ_1_^+^). Under this *irrep*, the magnetic moment on the atoms related by the centering translation (1/2 1/2 0) invert their sign. This symmetry describes an AF ordering between the 4 Co^2+^ atoms in the unit cell and does not allow any ferromagnetic component (see the Fourier coefficients in Table 3). The magnetic space group is *P*2/*c*.1′*c* [*C*2/*c*] (No. 13.74).

The best adjustment of the Rietveld refinement for the 1.6 K pattern is reached with m_*x*_ = −m_*z*_ = 1.491 (2) and m_*y*_ = 0 describing magnetic moments along the (1 0 1̅) direction, with a modulus of 2.1 (3) μ_B_. The magnetic structure of **4** can be explained as 2D ferromagnetic layers along the bc plane (with magnetic moments pointing along the (1 0 1̅) axis and in concordance with the lamellar Co/μ_4_-HCOO substructure. These ferromagnetic layers are coupled antiferromagnetically along the *a*-axis (see Figure 12B and Figure S11).

In agreement with its manganese isomorph, the Co^2+^ ion in compound **4** also has a magnetic moment with components in the *a* and *c* axes, arising from an AF arrangement in the Co/μ_4_-HCOO substructure. However, in this case, the layers acquire a ferromagnetic arrangement between each other, as the magnetic moments of the cations along the *b*-axis possess the same orientation.

The difference between the magnetic structures of **3** and **4** can be explained if one considers the arrangement of the formate bridges and the magnetic orbitals of manganese and cobalt cations. In the case of **3**, the manganese ion possesses its five *d* orbitals with a magnetic character, so it is difficult to establish a magneto-correlation between the carboxylate bridge conformation and the nature of the coupling pathway. This complex mixture of five magnetic orbitals and either both *syn–anti* and *anti–anti* conformations lead to a complicated competition between ferromagnetic and AF couplings, which are probably suppressing each other and consequently explaining the low *zJ* value obtained from the asymptotic Curie temperature (Table 2).

In contrast to the previous situation, compound **4** has two of the three magnetic orbitals belonging to the *e*_*g*_ level. The magnetic orbital distribution can be approximated to the scheme of Figure 1. In this case, the *syn–anti* layers induce ferromagnetic ordering, while the *anti–anti* bridges cause AF interactions, in agreement with what is proposed for the carboxylate linking configurations.^29−32^

**Figure 14:**
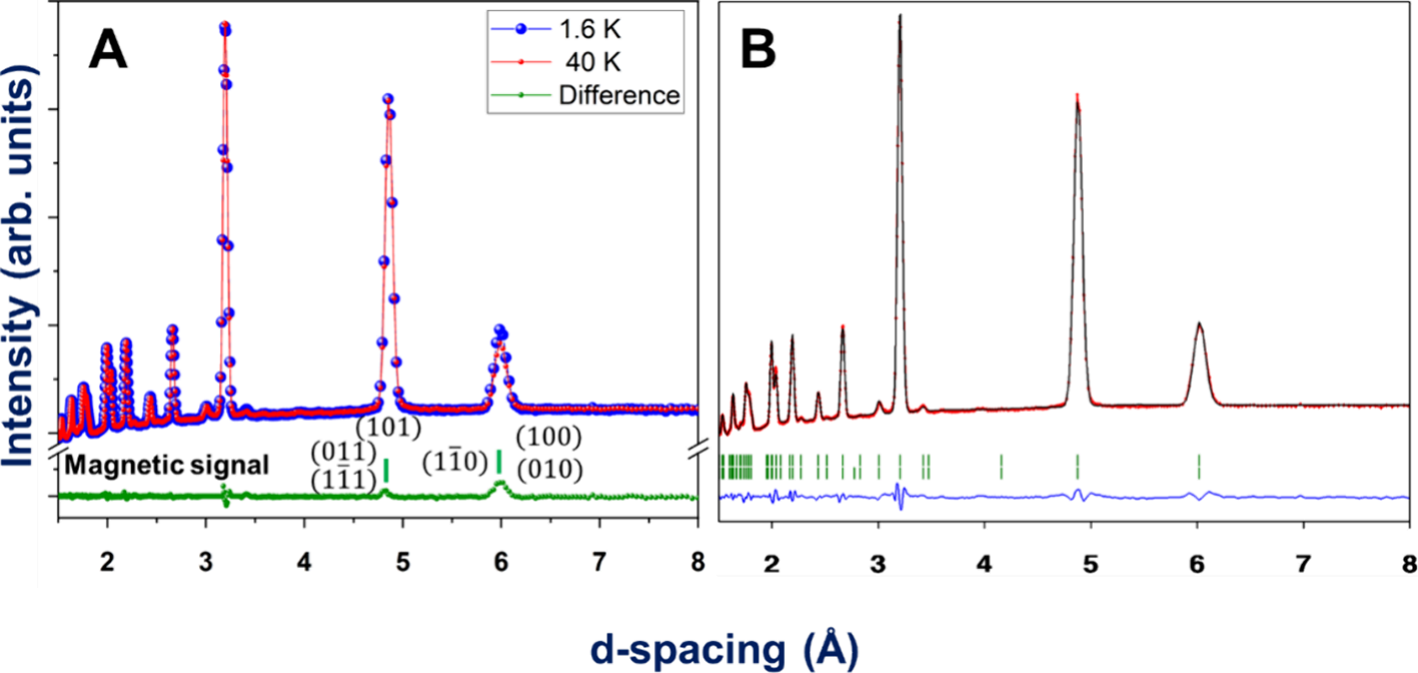
For compound **5.** (A) Diffractograms measured at 40 K (red) and 1.6 K (blue) and differences (green). The magnetic signal is indexed with **k** = (0 0 0). (B) Rietveld refinement for the 1.6 K diffractogram. The experimental points are in red, the fit is the black continuous line, and the difference between data and fit is the blue line. The green lines are signaling the positions of the Bragg lines.

Diffractograms at the paramagnetic phase (30 K) and the ordered phase (1.6 K) were collected in the DMC instrument using a constant wavelength of 2.45 Å for compound **5.** At low temperatures, no new peaks are observed, but just a small increase of intensity of the large *d*-spacing (low angle) Bragg’s lines is evidenced (Figure 14A), affording us to assume a propagation vector **k** = (0 0 0). For the Ni^2+^ cation at the 2d Wyckoff position of the *P*6_3_22 space group, the magnetic representation is decomposed as a direct sum as expressed in Table 3 and Table S7.

The low-temperature pattern was fitted by mΓ_3_ (in red in Table 3), which allowed us to reach the best agreement. This *irrep* describes the magnetic moments in an AF ordering only along the *c* axis, which agrees with the negative value of theta from the susceptibility measurements.

**Figure 15:**
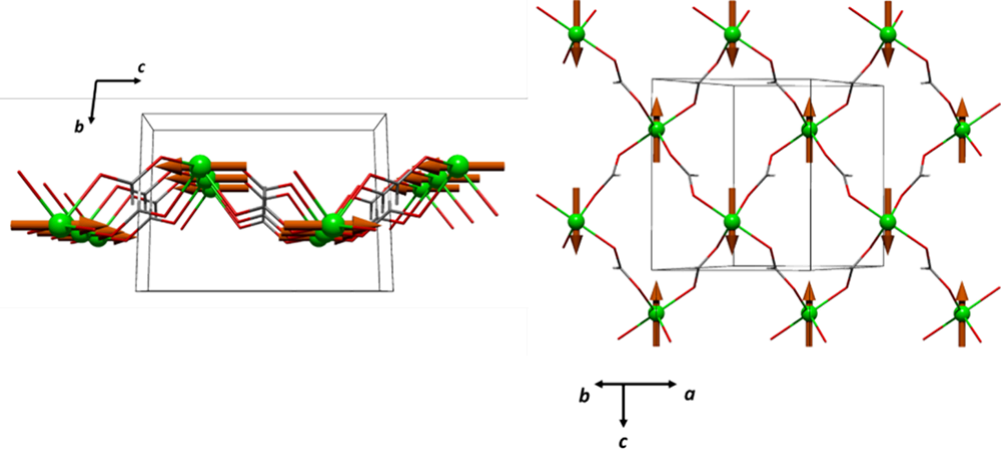
Magnetic structure for compound **5**.

Rietveld refinement (Figure 14B) delivers a magnetic moment of 1.47 (9) μ_B_ at 1.6 K, smaller than the expected value for an ion with *S* = 1 as Ni^2+^ (2.0 μ_B_). In agreement with compounds **3** and **4**, this AF ordering grows along the 2D subnet defined by the M_4_ Ni^2+^/HCOO units in the ac plane. Similar to compound **3**, these AF layers are ordered in an antiparallel fashion along the *b* axis, allowing the CP to acquire a global AF structure in concordance with the magneto-thermal analysis (Figure 15).

The antiparallel ordering inside the layers in compound **5** is in agreement with the proposed model about the influence of the carboxylate bridge on the magnetic coupling nature for *e_g_* magnetic orbitals. In this case, the *anti–anti* bridges inside layers promotes the AF interactions between the Ni^2+^, which have (*t*_2g_)^6^(*e_g_*)^2^ electronic configuration. In the same sense, the coupling between two ions from different layers also has an AF character as the formate bridge has also an *anti–anti* fashion.

The same observation can be made for compounds **1** and **2**, where only *anti–anti* bridges are present in the nuclear structure, both inside and between the layers. For both cases, we only see AF interactions between the ions, in agreement with the proposed model.

On the other hand, the magnetic symmetry determined in compounds **1**, **2**, and **3** with the neutron diffraction experiments, allows some ferromagnetic component along the *b-*axis, in agreement with their macroscopic magnetic measurements, whereas for compounds **4** and **5**, their magnetic structures are purely AF. However, the formates studied here, even if they do not have an inversion center, do not develop any incommensurate structure, like in the {[CH_3_NH_3_][Ni(HCOO)_3_]}_*n*_ derivative,^44^ or helical structure like in the [Co_3_(HCOO)_5_Cl(H_2_O)_2_]_*n*_.^45^ Moreover, the compounds here described cannot host any chiral soliton lattice, like the one anticipated for the {[NH_4_][Mn(HCOO)_3_]}_*n*_ compound.^44^

## Conclusions

To determine the ordered arrangement of the magnetic spin within 3D lattices, we synthesized five transition metal formate CPs, three of which were synthesized here for the first time, [NaCo(HCOO)_3_(H_2_O)_2_]_*n*_ (**1**), [NaNi(HCOO)_3_(H_2_O)_2_]_*n*_ (**2**), and [KNi(HCOO)_3_]_*n*_ (**5**), crystallizing in Sohncke space groups. A complete structural, magnetic, and thermal characterization has been done, allowing us to obtain the nuclear structure and determine that compounds **1**, **2**, and **5** present chiral structures, unlike the compounds [KMn(HCOO)_3_]_*n*_ (**3**) and [KCo(HCOO)_3_]_*n*_ (**4**). Interestingly, only in compounds **3** and **4**, the 2D subnet formed by the 3d cations bridged by formate anions are in a *syn–anti* coordination mode. In the five 3D networks, AF interactions are predominant, and the estimation of the Neél temperatures, which are in good agreement with the maximum observed through the heat capacity measurements, permits us to infer the existence of magnetic long-range ordering in the reported lattices.

Neutron diffraction in powders and single crystals at different temperatures in the ordered and paramagnetic phases allowed information on the magnetic structure for each studied lattice, including the values of the transition metal-ordered magnetic moments. The magnetic structures of each compound at low temperatures have been classified according to the *irreps* theory, and the magnetic space groups have also been assigned. The compounds **1**, **2**, **3**, and **5** are characterized by presenting AF interactions through the 2D substructure, while the interaction between the lamellas are AF for **1** and **2** and FM for **3** and **5**. In contrast, compound **4** is characterized by an FM interaction within the 2D substructure (*bc* plane) and AF between the lamellas.

This fine crystallographic study and the complete description of the magnetic structure allowed us to understand the role of the different coordination modes of the formate ligand for the assembly of the 3D networks and to generate new materials where ferro- or AF interactions between the metallic cations could be present. Therefore, our approach to obtain antisymmetric magnetic interaction and therefore noncollinear magnetic structures by using formate chiral ligand has been validated through the determination of the magnetic structures of each compound.

## Supporting Information Available

Additional information on the refinement of single-crystal and powder diffraction data, supplementary magnetization measurements, complete description of the magnetic representations (PDF)
