# Supplementary material for: Magneto-Structural Correlations in Coordination Polymers Based on Formate Ligand and Transition Metal Cations
Source: Inorg Chem. 2025 Apr 10;64(19):9758–71. doi: 10.1021/acs.inorgchem.4c04737 (PMC12093373; doi:10.1021/acs.inorgchem.4c04737)
Supplement: Supplementary file 1 — ic4c04737_si_001.pdf [file ic4c04737_si_001.pdf]

Magneto-structural correlations in coordination polymers based on formate ligand and transition metal cations

Francisco Rubio-Sepúlveda[a,b,c], Alicia Manjón-Sanz[d], Laura Cañadillas-Delgado[e], José Alberto Rodríguez-Velamazán[e], Lukas Keller[f], Denis Sheptyakov[f], Diego Venegas-Yazigi[a,c], Verónica Paredes-García[c,g*], Javier Campo[b*]*

[a] Universidad de Santiago de Chile, Facultad de Química y Biología, Departamento de Química de los Materiales, Santiago 9170022, Chile.

[b] Instituto de Nanociencia y Materiales de Aragón (CSIC - Universidad de Zaragoza), Zaragoza 50009, Spain.

[c] Centro para el Desarrollo de la Nanociencia y Nanotecnología CEDENNA, Santiago, 9170022, Chile.

[d] Oak Ridge Natl Lab, Neutrons Scattering Div, Oak Ridge, TN 37831 USA.

[e] Institut Laue Langevin (ILL), CS 20156, 38042 Grenoble Cedex 9, France

[f] Laboratory for Neutron Scattering and Imaging, Paul Scherrer Institut, 5232 Villigen PSI, Switzerland.

[g] Universidad Andres Bello, Facultad de Ciencias Exactas, Departamento de Ciencias Químicas, Santiago 8370146, Chile.

Email: vparedes@unab.cl; diego.venegas@usach.cl; javier.campo@csic.es

Date: March 6th, 2025

SUPPLEMTARY FIGURES

Figure S1. Superposition of two lamellar substructures in compound **1**.

Figure S2. Hydrogen bonds between water molecule and formate ligands in compound **1**.

Figure S3. **A,** Asymmetric unit of compound **5**; **B,** *anti – anti* formate bridiging mode pointing the K – O – Ni triangles and **C,** view of the dihedral angle between the Ni–O–K triangles.

Figure S4. Susceptibility curves in the range of 2 – 300 K for compounds **1-5**.

Figure S5. Susceptibility curves in the range of 2 – 25 K for compound **1** using different values of applied magnetic field.

Figure S6. Susceptibility curves in the range of 20 – 40 K for compound **2** using different values of applied magnetic field.

Figure S7. Histersis loop at 2 K for compound 5. Range of ±90 kOe (left) and ± 0.8 kOe (right).

Figure S8. Isothermal magnetization plots at 2 K in the range of ±90 kOe for **A**, **4** and **B**, **5**.

Figure S9. Rietveld refinement of compound 1

Figure S10. Magnetic structure of $[KMn(HCOO)_3]_n$

Figure S11. Magnetic structure of $[KCo(HCOO)_3]_n$

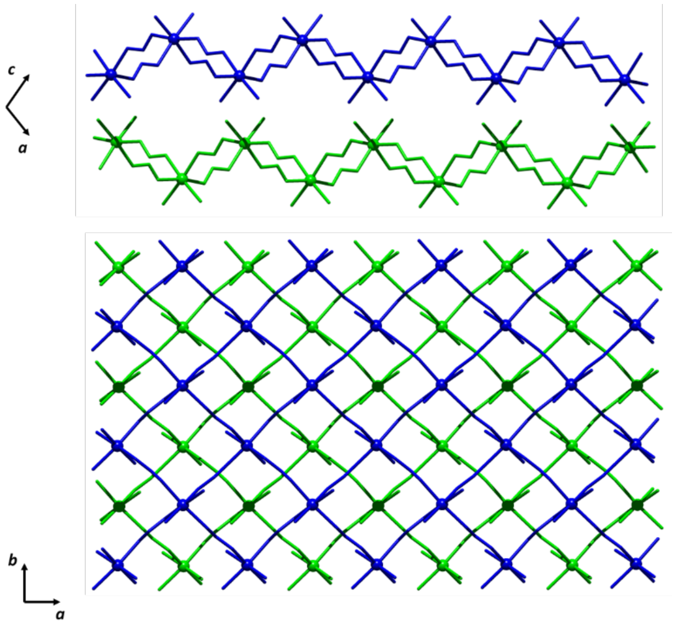

Figure S1

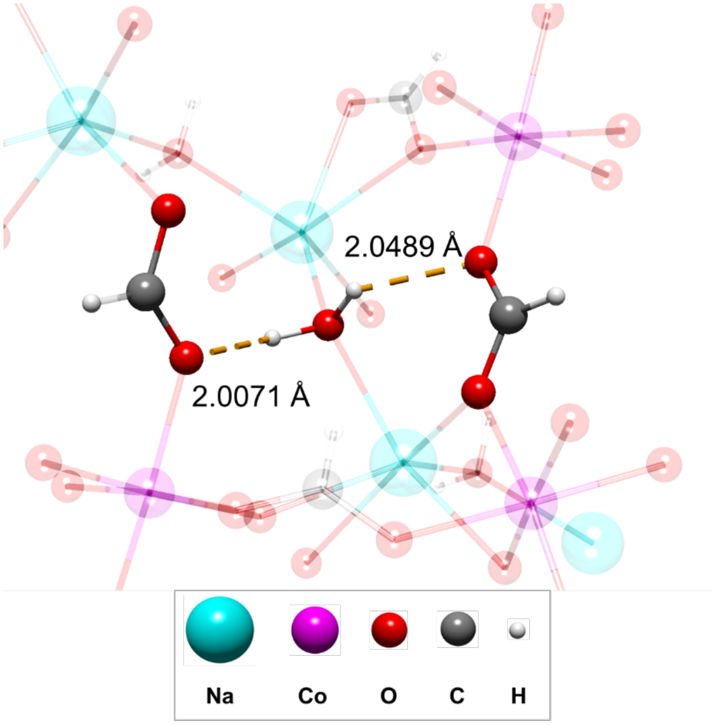

Figure S2.

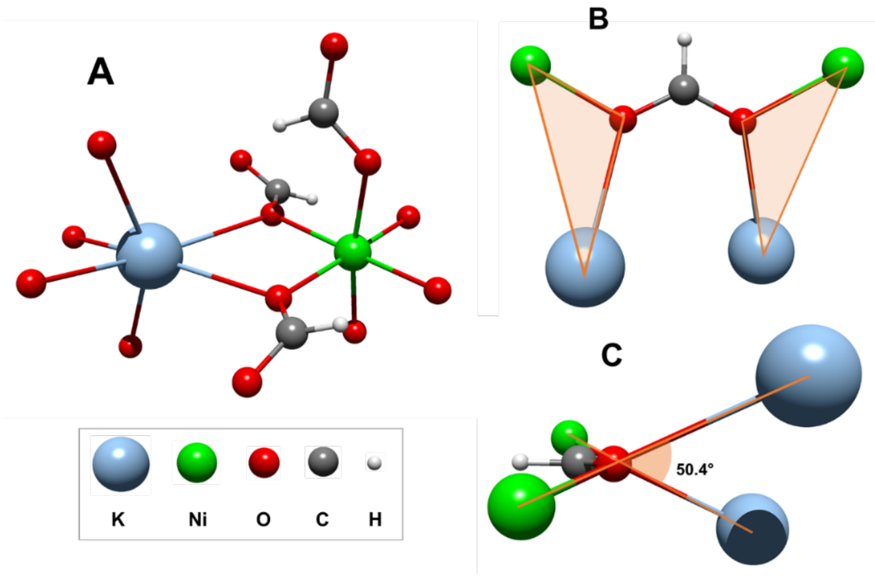

Figure S3

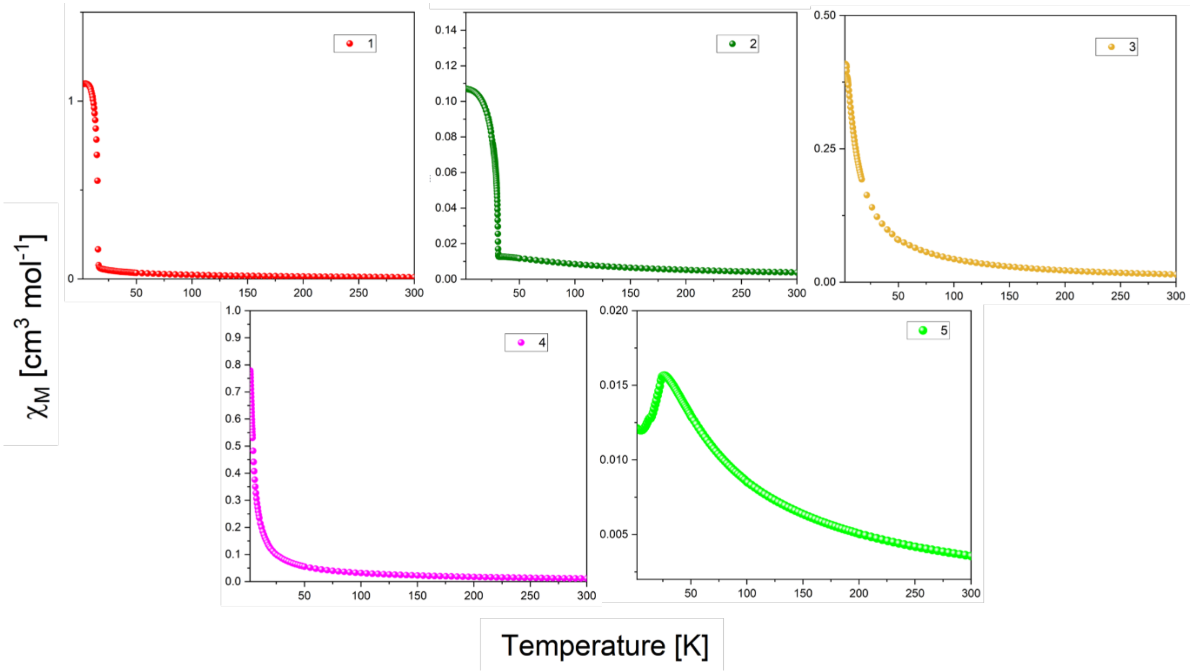

Figure S4

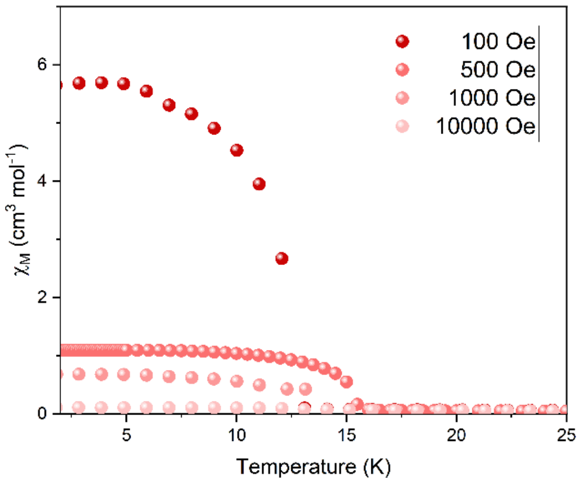

Figure S5

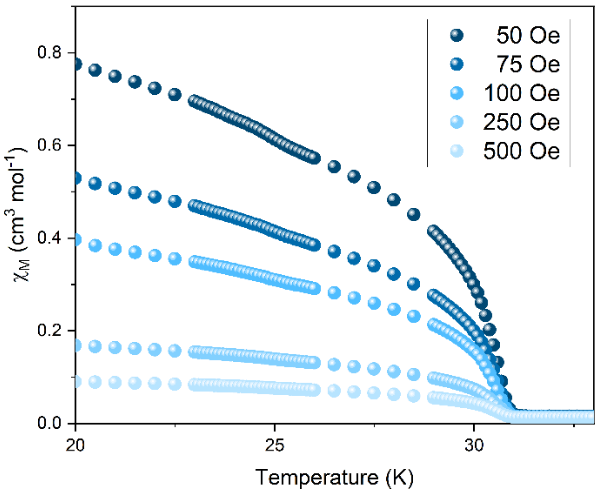

Figure S6

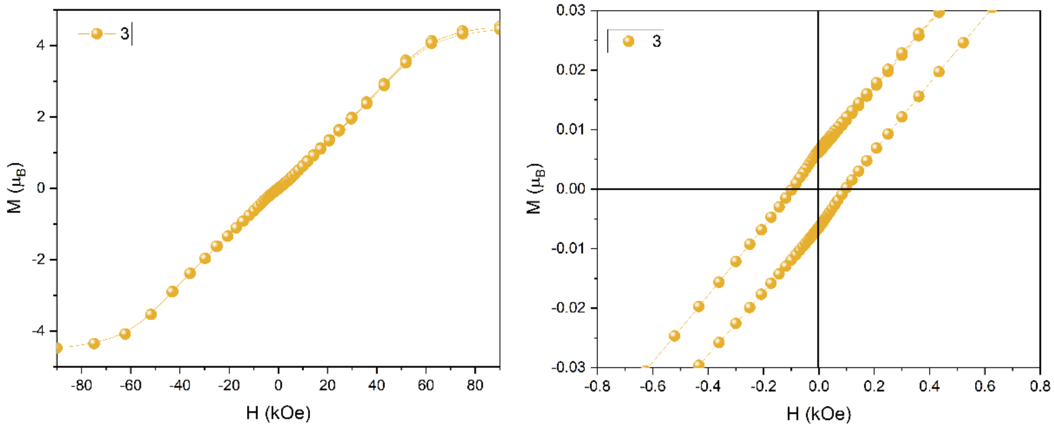

Figure S7

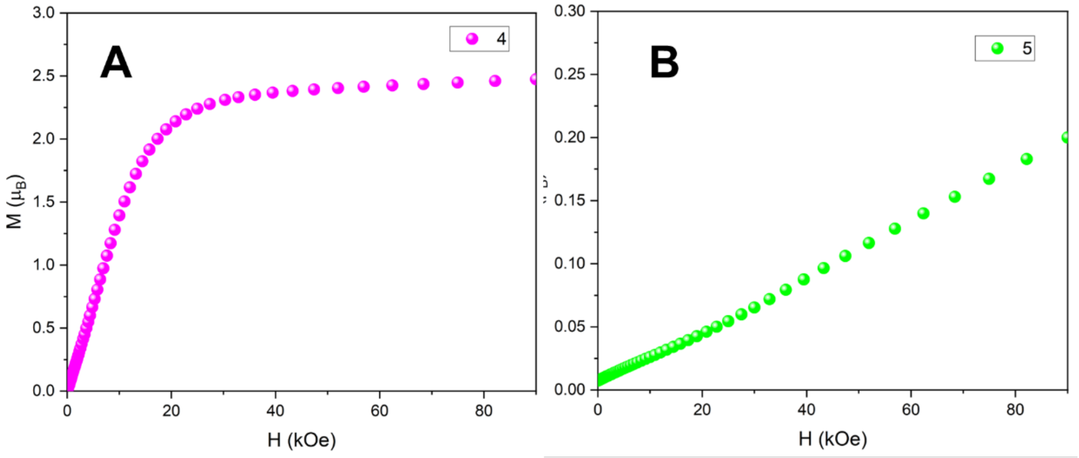

Figure S8

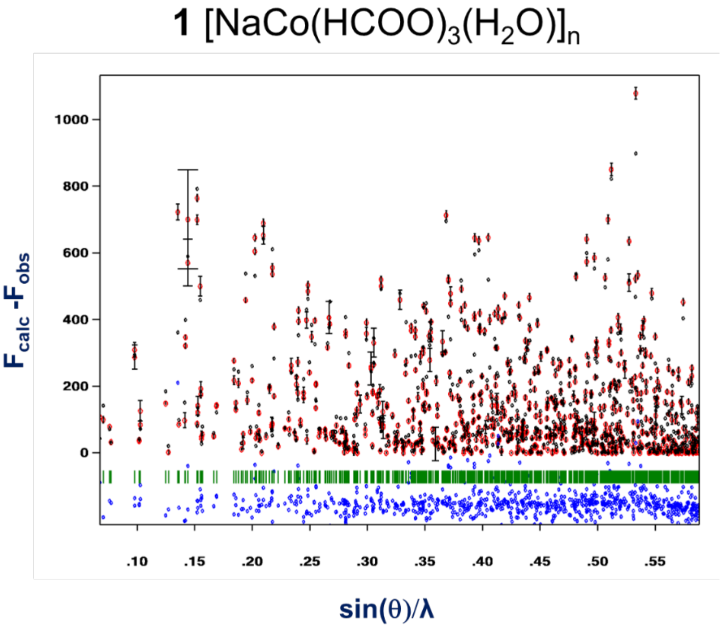

Figure S9

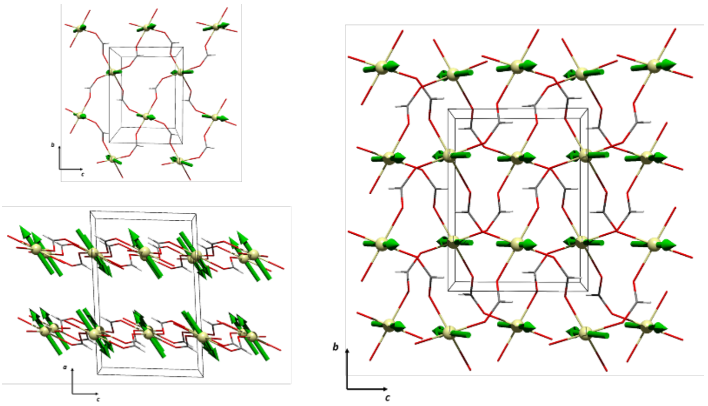

Figure S10

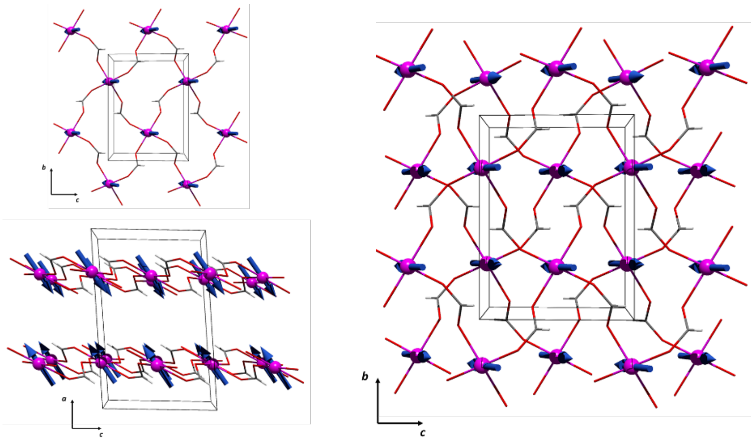

Figure S11

SUPLEMENTARY TABLES

**Table S1**. Syngle Crystal Neutron crystallographic data for compounds **1**.	
Compound	**1**
Formula	$[NaCo(HCOO)_3(H_2O)_2]_n$
MW [g mol^{-1}]	252.99
Space group	$P2_1$
Radiation	1.45 Å
*T [*K*]*	20
*a [*Å*]*	7.3369(8)
*b [*Å*]*	7.3864(9)
*c [*Å*]*	7.8995(9)
β [°*]*	116.475(7)
*V [*Å^3*]*	383.20(8)
Z	2
*ρ [*g cm^{-3}*]*	2.036
*μ [*mm^{-1}*]*	0.146
F(000)	96
sinΘ/λ	0.0677 – 0.5884
R_{int}	0.0813
R_1	0.0937
wR_2	0.1077
GoF	5.5

**Table S2**. Powder Neutron crystallographic data for compounds **2 – 5**.				
Compound	**2**	**3**	**4**	**5**
Formula	$[NaNi(HCOO)_3(H_2O)_2]_n$	$[KMn(HCOO)_3]_n$	$[KCo(HCOO)_3]_n$	$[KNi(HCOO)_3]_n$
MW [g mol^{-1}]	252.77	229.09	233.09	232.85
Space group	$P2_1$	$C2/c$	$C2/c$	$P6_322$
Radiation	2.45 Å	1.50 Å	1.88 Å	2.45 Å
*T [*K*]*	40	4	50	30
*a [*Å*]*	7.3312(4)	10.7794(6)	10.7941(1)	6.9468(1)
*b [*Å*]*	7.3138(7)	9.0876(5)	9.0457(1)	6.9468(1)
*c [*Å*]*	7.8155(5)	7.0000(3)	6.8553(1)	8.3210(5)
β [°]	116.745(4)	94.077(4)	94.647(9)	90
*V [*Å^3*]*	374.22(8)	683.98(6)	667.15(1)	347.76(2)
Z	2	4	4	2
*ρ [*g cm^{-3}*]*	2.083	2.225	2.320	2.224
sinΘ/λ	0.0107 – 0.3778	0.0592 – 0.5650	0.0176 – 0.4718	0.0107 – 0.3771
R_{Bragg}	8.10	4.81	4.48	3.90
R_F	5.29	14.7	3.01	5.72
GoF	3.4	0.33	2.7	4.9

**Table S3**. Bond Valence Sum for M^{2+} in compounds **1** – **5**.				
Compound	Cation	Oxidation State	BVS	Deviation
1	Co^{2+}	2+	2.03	+0.03
2	Ni^{2+}	2+	1.89	-0.11
3	Mn^{2+}	2+	2.11	+0.11
4	Co^{2+}	2+	1.99	-0.01
5	Ni^{2+}	2+	1.98	-0.02

**Table S4**. *Irreps*, Fourier coefficient and basis vectors for $P2_1$ space group with **k** = (0 0 0) and WP(2a)						
	{1|000}<br>(x, y, z)			{2_{0y0}|0½0}<br>(-x,1/2+y,-z)		
irreps	#1	$\mathbf{S_k}^{\mathbf{1}}$	Basis vectors	#2	$\mathbf{S_k}^{\mathbf{2}}$	Basis vectors
mΓ_1	1	(u, v, w)	(1, 0, 0), (0, 1, 0), (0, 0, 1)	1	(-u, v, -w)	(-1, 0, 0), (0, 1, 0), (0, 0, -1)
mΓ_2	1	(u, v, w)	(1, 0, 0), (0, 1, 0), (0, 0, 1)	-1	(u, -v, w)	(1, 0, 0), (0, -1, 0), (0, 0, 1)

Table S5. *Irreps*, Fourier coefficient and basis vectors for *C*2/*c* space group with **k** = (0 0 0) and WP(4c)

	{1|000} (x, y, z)			{2_{0y0}|00¼} (-x, y, -z+½)			{-1|000} (x+½, y+½, z)			{m_{x0z}|00½} (-x+½, y+½, -z+½)		
Irreps	#1	$\mathbf{S_k}^1$	Basis vectors	#2	$\mathbf{S_k}^2$	Basis vectors	#3	$\mathbf{S_k}^3$	Basis vectors	#4	$\mathbf{S_k}^4$	Basis vectors
$m\Gamma_1^+$	1	(u,v,w)	(1, 0, 0), (0, 1, 0), (0, 0, 1)	1	(-u,v,-w)	(1, 0, 0), (0, 1, 0), (0, 0, 1)	1	(u,v,w)	(1, 0, 0), (0, 1, 0), (0, 0, 1)	1	(-u,v,-w)	(1, 0, 0), (0, 1, 0), (0, 0, 1)
$m\Gamma_2^+$	1	(u,v,w)	(1, 0, 0), (0, 1, 0), (0, 0, 1)	-1	(u,-v,w)	(1, 0, 0), (0, 1, 0), (0, 0, 1)	1	(u,v,w)	(1, 0, 0), (0, 1, 0), (0, 0, 1)	-1	(u,-v,w)	(1, 0, 0), (0, 1, 0), (0, 0, 1)

Table S6. *Irreps*, Fourier coefficient and basis vectors for *C*2/*c* space group with **k** = (0 1 0) and WP(4c)

	{1|000} (x, y, z)			{2_{0y0}|00¼} (-x, y, -z+½)			{-1|000} (x+½, y+½, z)			{m_{x0z}|00½} (-x+½, y+½, -z+½)		
Irreps	#1	$\mathbf{S_k}^1$	Basis vectors	#2	$\mathbf{S_k}^2$	Basis vectors	#3	$\mathbf{S_k}^3$	Basis vectors	#4	$\mathbf{S_k}^4$	Basis vectors
mY_1^-	1	(u,v,w)	(1, 0, 0), (0, 1, 0), (0, 0, 1)	-1	(-u,v,-w)	(1, 0, 0), (0, 1, 0), (0, 0, 1)	-1	(u,v,w)	(1, 0, 0), (0, 1, 0), (0, 0, 1)	-1	(-u,v,-w)	(1, 0, 0), (0, 1, 0), (0, 0, 1)
mY_2^-	1	(u,v,w)	(1, 0, 0), (0, 1, 0), (0, 0, 1)	-1	(u,-v,w)	(1, 0, 0), (0, 1, 0), (0, 0, 1)	-1	(u,v,w)	(1, 0, 0), (0, 1, 0), (0, 0, 1)	1	(u,-v,w)	(1, 0, 0), (0, 1, 0), (0, 0, 1)

Table S7. *Irreps*, Fourier coefficient and basis vectors for *P*$6_3$22 space group with **k** = (0 0 0) and WP(2d)

	{1|000} (x, y, z)			{2_{00z}|00½} (-x, -y, z+½)		
Irreps	#1	$\mathbf{S_k}^1$	Basis vectors	#1	$\mathbf{S_k}^3$	Basis vectors
$m\Gamma_2$	1	(0,0,w)	(0, 0, 0), (0, 0, 0), (0, 0, 1)	1	(0,0,w)	(0, 0, 0), (0, 0, 0), (0, 0, 1)
$m\Gamma_3$	1	(0,0,w)	(1, 0, 0), (0, 1, 0), (0, 0, 1)	-1	(0,0,-w)	(1, 0, 0), (0, 1, 0), (0, 0, -1)
$m\Gamma_5$	$\begin{pmatrix}1 & 0\\0 & 1\end{pmatrix}$	($r_{0.u-v}$,u,0)	(0.5, 1, 0)<br>(-1, 0, 0)	$\begin{pmatrix}1 & 0\\0 & 1\end{pmatrix}$	(-$r_{0.u+v}$,-u,0)	(-0.5, -1, 0)<br>(1, 0, 0)
$m\Gamma_6$	$\begin{pmatrix}1 & 0\\0 & 1\end{pmatrix}$	($r_{0.u-v}$,u,0)	(0.5, 1, 0)<br>(-1, 0, 0)	$\begin{pmatrix}-1 & 0\\0 & -1\end{pmatrix}$	($r_{0.u-v}$,u,0)	(0.5, 1, 0)<br>(-1, 0, 0)
